# Effects of Acute Carbohydrate–Electrolyte Supplementation on Exercise Performance in Moderate-to-Hot Ambient Conditions: A Systematic Review and Meta-Analysis

**DOI:** 10.3390/nu18193200

**Published:** 2026-09-28

**Authors:** Xinkai Wang, Taihe Liang, Yiran Liu, Xuda Zhang, Shiao Zhao, Zhide Liang, Senyao Du, Yixiang Peng, Ziheng Ning

**Affiliations:** Faculty of Health Sciences and Sports, Macao Polytechnic University, Rua de Gomes, Macao 999078, Chinatigerleung4177@gmail.com (T.L.); p2417052@mpu.edu.mo (Y.L.); p2417093@mpu.edu.mo (X.Z.); p2314096@mpu.edu.mo (S.Z.); liangzhide123@163.com (Z.L.); dusenyao22@163.com (S.D.); p2525351@mpu.edu.mo (Y.P.)

**Keywords:** sports nutrition, endurance capacity, hydration strategy, thermal strain, ergogenic support, multilevel evidence synthesis

## Abstract

Background: An ambient-temperature eligibility criterion of ≥22 °C was treated as an operational lower boundary spanning moderate or mildly warm to hot conditions, not as a universal physiological threshold for heat stress. This systematic review and three-level meta-analysis evaluated acute carbohydrate–electrolyte (CHO-E) supplementation versus a water or non-caloric placebo during exercise at these temperatures. Methods: Following PROSPERO registration (CRD420261410953), five databases were searched to May 2026 for randomized controlled trials. Nested effect sizes were synthesized using three-level random-effects models (Hedges’ g), and certainty was evaluated with RoB 2 and GRADE. Results: A secondary omnibus synthesis included 32 effect sizes from 21 studies (N = 316) and yielded a small positive average estimate (g = 0.37, 95% CI: 0.21 to 0.53; *p* < 0.001; *I*^2^ = 46.7%), although the 95% prediction interval crossed the null (−0.21 to 0.96). Primary outcome-domain analysis identified a positive average estimate for aerobic endurance (g = 0.45, 95% CI: 0.26 to 0.64), whereas evidence for anaerobic endurance, explosive power, and strength remained inconclusive. Post hoc threshold sensitivity analyses retained positive mean estimates after excluding trials at 22–25 °C (>25 °C: g = 0.41, 95% CI: 0.23 to 0.59) and at ≥28 °C (g = 0.36, 95% CI: 0.11 to 0.61), but both prediction intervals crossed zero. Neither ambient temperature nor exposure duration was a significant linear moderator. Conclusions: Acute CHO-E supplementation was associated with a small average performance difference, concentrated in aerobic endurance, within the specified ambient-temperature range. Because the trials compared treatments within the same environmental condition rather than hot with thermoneutral exercise, these results do not establish that CHO-E offsets heat-induced performance decrements.

## 1. Introduction

Exercise performance is a central outcome in sports science, exercise physiology, and applied sports nutrition, reflecting the integrated capacity of the human body to sustain energy production, regulate fatigue, maintain fluid and electrolyte balance, and tolerate physiological stress during physical activity. In competitive sport, military training and operations, occupational tasks, and recreational exercise, the ability to preserve performance during prolonged or high-intensity activity has substantial practical relevance for athletes, military personnel, practitioners, and physically active individuals. From the perspective of sports nutrition, nutrient provision and hydration-related strategies before and during exercise represent modifiable, field-practical approaches that may influence substrate availability, hydration status, fatigue development, and the capacity to sustain exercise intensity. Therefore, sports nutrition is not merely a supportive practice for performance enhancement, but also an important scientific framework for understanding how exogenous substrate and hydration support interact with physiological demands during exercise. The significance of this issue is further amplified by the continuing rise in global ambient temperatures, which increases the likelihood that training, competition, military operations, and outdoor physical activity will occur under warm or hot environmental conditions [1,2]. Because heat exposure can impose additional thermoregulatory and cardiovascular strain, accelerate fluid loss, and compromise the maintenance of prolonged exercise intensity, identifying effective nutritional strategies under elevated ambient temperature conditions has become both a timely practical priority and a scientifically important question in exercise nutrition research.

Within this framework, carbohydrate supplementation constitutes a central nutritional approach for supporting exercise performance under conditions of increased physiological demand [3]. Maintenance of carbohydrate availability is mechanistically important because endogenous carbohydrate stores may decline during sustained or metabolically demanding exercise, and insufficient carbohydrate availability can contribute to fatigue development, impaired exercise capacity, and reduced external work production [4,5]. Physiologically, ingested carbohydrate can be absorbed and utilized as an exogenous substrate, contributing to carbohydrate-dependent energy metabolism and helping to maintain circulating glucose availability during exercise. In this way, carbohydrate ingestion may attenuate dependence on endogenous glycogen stores and support the continuation of high rates of carbohydrate metabolism [5,6]. Collectively, these mechanisms provide a physiological basis for the role of carbohydrate supplementation in supporting exercise performance across diverse exercise tasks. Heat exposure can also increase carbohydrate oxidation and accelerate glycogen use at a given absolute workload, providing an additional rationale for maintaining exogenous carbohydrate availability during prolonged exercise [7].

Electrolytes also have important mechanistic relevance during exercise, particularly under warm or hot environmental conditions. Prolonged exercise, repeated high-intensity activity, and heat exposure can increase sweat loss and fluid turnover, thereby challenging fluid–electrolyte homeostasis and adding to cardiovascular and thermoregulatory strain [1,8]. Major electrolytes, including sodium, potassium, and chloride, are involved in osmotic regulation, extracellular and intracellular fluid distribution, neuromuscular function, and muscle contraction during exercise [9]. Accordingly, electrolyte provision during exercise may help support fluid–electrolyte regulation, fluid retention, and hydration-related physiological stability, thereby providing a physiological basis for maintaining exercise performance when sweat losses are elevated [3]. Co-delivery of carbohydrate, fluid, and sodium may support intestinal glucose–sodium transport, beverage retention, and substrate delivery, although the included comparisons cannot separate these components experimentally [3,8].

Although carbohydrate–electrolyte (CHO-E) supplementation is supported by a coherent mechanistic rationale [5,8], the extent to which it is associated with measurable performance differences when exercise is performed at ambient temperatures of ≥22 °C remains insufficiently established. Available studies report direct performance outcomes across a continuum of environmental conditions and exercise tasks, with some showing benefits and others showing no whole-trial improvement [10,11,12]. A ≥22 °C boundary was part of the eligibility criteria in the originally submitted review and was used as a broad operational lower boundary rather than as a universal physiological threshold for heat stress; the numeric cutoff was not enumerated in the PROSPERO registration record. A prior heat-mitigation synthesis used conditions above 20 °C as a broad warm-or-hot evidence base [13], but experimental work shows that endurance capacity can vary across temperatures in the low 20s °C range without identifying a single threshold [14], and 22 ± 1 °C has also been described as a moderate environment in another exercise study [15]. Thermal strain therefore cannot be inferred from dry-bulb temperature alone and also depends on humidity, air velocity, radiant heat, clothing, metabolic heat production, exposure duration, and individual characteristics [1]. Differences in exercise mode and duration, training status, environmental severity, acclimation, beverage concentration, fluid dose, timing, and control composition are plausible sources of inconsistent study-level findings.

Accordingly, this systematic review and meta-analysis aimed to estimate the difference in direct exercise performance between acute CHO-E supplementation and water or non-caloric placebo during exercise conducted at reported ambient temperatures of ≥22 °C. The included trials compared treatments within the same environmental condition; they did not compare the same treatment under hot versus thermoneutral conditions. The review therefore evaluates CHO-E-associated performance differences within the specified temperature range and does not estimate a heat-specific protective interaction.

## 2. Methods

This systematic review and meta-analysis was registered in the International Prospective Register of Systematic Reviews (PROSPERO; CRD420261410953) after completion of database searching and before study screening, data extraction, risk-of-bias assessment, and quantitative synthesis, as recorded in the registration-stage fields. No separate full protocol was prepared beyond the PROSPERO registration record. The review was conducted and reported in accordance with the Preferred Reporting Items for Systematic Reviews and Meta-Analyses (PRISMA) 2020 statement [16], and the completed checklist is provided in Supplementary Material S2. The PROSPERO record identified endurance-related or sustained exercise performance as the primary objective and listed time to exhaustion, time-trial completion time, mean power, total work, and distance as main outcomes.

The implemented review differed from the PROSPERO record in four respects that are disclosed here for transparency. First, the final implemented database set was PubMed, Embase, Web of Science Core Collection, CENTRAL, and Scopus, whereas the registration record listed an intended source set that also included the Cochrane Database of Systematic Reviews and Google Scholar but did not list Web of Science. Second, the implemented review applied an English-language restriction, whereas the registration record stated no language restriction. Third, the operational ≥22 °C eligibility boundary used in the originally submitted review was not numerically specified in the registration record. Fourth, the four outcome-domain categories were used in the original submission, but the revised primary interpretive emphasis on construct-specific estimates and the secondary status of the cross-domain omnibus summary were introduced during revision. Fifth, the eligibility wording was clarified during revision to include complete randomized primary reports published as journal articles or formal scholarly proceedings papers when methods and extractable performance data were available; conference abstracts without sufficient methodological and numerical detail remained ineligible.

The four outcome-domain categories were already used in the originally submitted analysis. However, the reporting hierarchy that gives construct-specific estimates—particularly aerobic endurance—primary interpretive priority and presents the cross-domain estimate as a secondary omnibus summary was introduced during revision. The strict time-trial versus time-to-exhaustion comparison was also added post hoc.

### 2.1. Search Strategy

A systematic literature search was conducted from database inception in five sources: PubMed (NCBI PubMed), Embase (Embase.com, Elsevier), Web of Science Core Collection (Clarivate), the Cochrane Central Register of Controlled Trials (CENTRAL; Cochrane Library), and Scopus (Elsevier). PubMed, Embase, and Web of Science were searched on 27 May 2026, whereas CENTRAL and Scopus were searched on 28 May 2026. The original strategy used free-text terms, truncation, and Boolean operators and deliberately combined broad concept blocks for carbohydrate–electrolyte supplementation and exercise/performance without a separate heat, temperature, WBGT, or heat-stress block, so that potentially eligible studies would not be lost because environmental conditions were incompletely indexed. Warm-to-hot exposure (ambient temperature ≥22 °C) was therefore applied manually as an eligibility criterion during title/abstract and full-text screening rather than as a database restriction. To improve database-specific reporting, the original concept blocks were retrospectively mapped in Supplementary Material S1 to database-appropriate field tags and controlled-vocabulary syntax (MeSH for PubMed and CENTRAL, Emtree for Embase, TS= for Web of Science, and TITLE-ABS-KEY for Scopus). These reporting adaptations were not rerun; the result counts in S1 are those returned by the original searches on the dates stated. No publication-year restriction was applied. Reference lists of relevant reviews were also screened manually.

### 2.2. Eligibility Criteria

For this systematic review and meta-analysis, studies were included if they met the following inclusion criteria: (1) randomized controlled primary reports published as full journal articles or formal scholarly conference-proceedings papers with sufficient methods and extractable numerical data; (2) involved healthy, physically active individuals, athletes, or military personnel with a study-reported mean age of ≥15 years; when a numerical mean was not reported, an explicitly adult study sample was eligible; (3) eligible supplementation timing included pre-exercise, intra-exercise, or an inter-trial recovery interval preceding a subsequent performance assessment; (4) compared CHO-E supplementation with plain water or a non-caloric placebo or flavored-water control that did not provide physiologically active amounts of carbohydrate or electrolytes; (5) exercise was performed at a quantitatively reported ambient temperature of ≥22 °C, treated as an operational lower boundary rather than a universal heat-stress threshold; and (6) reported at least one extractable direct exercise-performance outcome.

For this review, acute supplementation was operationally defined as CHO-E administered within a single experimental session before exercise, during exercise, or during an inter-trial recovery interval preceding a subsequent performance test. Multi-day or chronic loading protocols, repeated supplementation extending beyond one experimental session, and post-exercise recovery protocols without a subsequent same-session performance assessment were classified as non-acute.

Studies were excluded if they met any of the following criteria: (1) non-randomized controlled studies, single-arm pre–post studies, observational studies, case reports, reviews, editorials, trial protocols, conference abstracts without sufficient methods or extractable data, animal studies, or in vitro studies; (2) the reported study-level mean age was <15 years, or participants had clinical or other conditions likely to substantially alter substrate use, fluid balance, or electrolyte metabolism; (3) the intervention was carbohydrate only, electrolytes only, a carbohydrate mouth rinse, of unclear composition, or co-administered with another independent ergogenic aid; (4) supplementation occurred only after exercise without a subsequent performance test; (5) quantitative ambient temperature was unavailable or <22 °C; (6) no extractable direct exercise-performance outcome was reported; (7) methodological detail was insufficient to assess eligibility; or (8) the report was not in English.

### 2.3. Study Selection Procedures

All retrieved records were imported into EndNote Version 21 [17] for duplicate removal. The 91,099 deduplicated records were then imported into ASReview LAB version 3.0.7 [18], which was used only to prioritize the order of manual screening and did not make inclusion decisions. Before iterative screening, a 26-record prior-knowledge set (8 relevant and 18 irrelevant) was created through keyword-assisted searching within the deduplicated library and independently judged by two reviewers (X.W. and T.L.) against the review PICOS criteria. The eight relevant priors were retained within the final count of 135 relevant records. The default ELAS u4 learner and unchanged default prioritization settings were used; no custom model or query strategy was applied. Two reviewers independently assessed prioritized records, reached consensus before final labels were entered, and resolved disagreements through discussion or, when necessary, adjudication by a third reviewer (Y.L.). Following the SAFE stopping framework [19], a threshold of 300 consecutive irrelevant records was specified before screening; 300 rather than 200 was selected to apply a more conservative stopping threshold. At stopping, 611 records had received human labels (135 relevant and 476 irrelevant, including the 26 priors).

The 135 records labeled relevant in ASReview were exported to Zotero [20] for independent manual title/abstract verification. Fifty-two records were removed at this stage, comprising 7 residual duplicates and 45 records that did not meet the eligibility criteria, leaving 83 reports sought for retrieval. Full texts could not be obtained for 52 reports, and the remaining 31 reports were independently assessed in full by X.W. and T.L. Disagreements at each manual screening stage were resolved through discussion or, if consensus could not be reached, by Y.L.

### 2.4. Data Extraction

Data extraction was independently performed by two reviewers (X.W. and T.L.) and organized using Microsoft Excel (Version 2019).

Data extracted included:

(1) Study characteristics: authors, publication year, study design (parallel or crossover);
(2) Participant characteristics: sample size, sex, age, training status, and health status;
(3) Intervention characteristics: type of CHO-E, dosage, supplementation duration and timing relative to exercise;
(4) Exercise-testing protocols, supplementation protocols, and reported performance findings;
(5) Direct exercise-performance outcomes specified in the eligibility and extraction framework: time-trial performance, time to exhaustion, power output, total work, distance completed, sprint or repeated-sprint performance, and intermittent exercise capacity;
(6) Environmental characteristics, including ambient temperature and heat-exposure duration.

For missing numerical data, the reviewers rechecked full texts, tables, supplementary files, registry records, and available author-provided materials. Authors of four studies were contacted, and replies were received for two studies. Four outcome datasets reported graphically were digitized using WebPlotDigitizer (version 5.2) [21]. X.W. and T.L. independently cross-checked the author-provided and digitized values against the available source materials and resolved discrepancies by discussion or consultation with Y.L.

Intervention concentration, fluid volume, timing, and electrolyte composition were coded descriptively when reported. Because these characteristics were incompletely reported, strongly correlated, and represented by few independent studies within many categories, no multivariable dose–timing model was fitted; the review cannot identify an optimal formulation, dose, volume, or timing strategy.

### 2.5. Risk of Bias

Risk of bias was reassessed for each eligible direct-performance result using the revised Cochrane risk-of-bias tool for randomized trials (RoB 2) [22]. The 20 crossover trials were evaluated with the dedicated crossover version, and the cluster-assigned parallel trial by Criswell et al. was evaluated with the cluster-randomized version, including the timing of participant identification and recruitment [23]. The domains were bias arising from the randomization process; for crossover trials, bias arising from period and carryover effects; bias due to deviations from intended interventions; bias due to missing outcome data; bias in measurement of the outcome; and bias in selection of the reported result. Signaling questions were answered as yes, probably yes, probably no, no, no information, or not applicable, and the decision algorithms were then used to assign low risk, some concerns, or high risk. For the study-level summary the least favorable judgment across eligible performance results and domains was used.

Allocation concealment was not inferred from terms such as ‘double blind’; it was coded as no information unless the report described a protected allocation process. Period and carryover risk was judged from sequence balance, modeling of period effects when required, and whether sufficient time elapsed before the second-period outcome, rather than from crossover design itself. Two reviewers (X.W. and T.L.) independently reassessed the full reports, additional source materials for the included studies, and available registry information; disagreements were resolved by discussion or third-reviewer adjudication (Y.L.). Study-level signaling-question responses, report-based rationales, and final judgments are provided in Supplementary Material S9.

The PEDro scale was retained only as a secondary descriptive reporting checklist [24], not as a second risk-of-bias instrument. Two reviewers (X.W. and T.L.) independently re-audited the 21 included reports and awarded a point only when the corresponding criterion was clearly supported by a literal reading of the report. PEDro formally awards a point or no point; NR was used in our audit table only as a transparency label for insufficient reporting and received no point. Eligibility (item 1) was recorded but excluded from the 10-point total, and generic wording such as ‘double blind’ was not treated as evidence that providers or outcome assessors were blinded. Disagreements were resolved by discussion or third-reviewer adjudication (Y.L.). The PEDro checklist was not used to modify the RoB 2 or GRADE judgments.

### 2.6. Statistical Analysis

#### 2.6.1. Effect-Size Calculation and Effect Measures

Effect-size calculations were conducted in accordance with the Cochrane Handbook for Systematic Reviews of Interventions (2nd edition, 2019) [25]. Because eligible outcomes were reported using different measurement scales across studies and most included trials had relatively small sample sizes, standardized mean differences were expressed as Hedges’ g with correction for small-sample bias [25].

The review question, eligibility criteria, and data-extraction framework specified a priori a broad set of direct exercise-performance outcomes. Their common feature was that each quantified externally observable performance or exercise capacity rather than a surrogate physiological, biochemical, or perceptual response. Hedges’ g was used to synthesize outcomes reported in different units, with all estimates aligned to a benefit-oriented direction. The cross-domain model was intended to provide an omnibus average across the eligible direct-performance evidence; it was not intended to imply that the test types are physiologically, psychometrically, or practically interchangeable.

Because the included studies comprised both within-subject crossover and parallel-group trials, effect sizes were calculated using design-specific methods. For crossover trials, the standardized mean difference was based on the average within-condition standard deviation (SMCRP), so that the effect-size denominator remained comparable with that used for independent-group standardized mean differences. The paired structure was incorporated through the within-participant correlation between the CHO-E and control conditions (r_pair) when calculating the effective degrees of freedom, Hedges’ small-sample correction, and sampling variance. For parallel-group trials, Hedges’ g was calculated from the between-group mean difference and pooled between-group standard deviation [25,26,27,28,29].

For eligible multi-arm trials involving more than one eligible intervention–comparator contrast sharing the same control condition, separate effect sizes were calculated for each relevant comparison. Shared control groups were not treated as independent studies, and their participant numbers were counted only once in descriptive summaries. Sample sizes from shared controls were not split. Dependence among multiple effect sizes from the same study was represented in a sampling variance–covariance matrix generated with metafor::vcalc. A separate sampling-error correlation parameter (ρ = 0.50) was used for this matrix. This parameter is conceptually distinct from the within-participant paired-condition correlation r_pair used in crossover effect-size calculations.

Because the included crossover trials did not report the within-participant correlation between CHO-E and control measurements, r_pair = 0.50 was used as a moderate working assumption for the primary analysis within the paired standardized-mean-difference framework described by Cousineau [28]. Because a single unreported correlation cannot be known exactly, sensitivity analyses repeated the complete crossover effect-size and sampling-variance calculations using r_pair = 0.20 and r_pair = 0.80, while the separate within-study dependence parameter ρ was held at 0.50. This varied only the paired-condition correlation assumption.

Effect sizes were interpreted using conventional thresholds: trivial (SMD < 0.20), small (0.20 ≤ SMD < 0.50), moderate (0.50 ≤ SMD < 0.80), or large (SMD ≥ 0.80) [30]. The specific equations used for these calculations are detailed below:

All 32 final effect sizes were calculated from condition-specific performance values and their reported condition-specific SDs. None used a pre–post change score; no change-score SD was reconstructed from separate pre- and post-exercise SDs, and no pre–post correlation was imputed. Accordingly, the only within-participant correlation assumption used in the effect-size calculations was the crossover paired-condition correlation r_pair described above.

For within-subject crossover trials, the average within-condition standard deviation was calculated as:

$${SD}_{\mathrm{av}}=\sqrt{\frac{{{SD}_{\mathrm{CHO}\text{-}E}}^{2}+{{SD}_{\mathrm{CON}}}^{2}}{2}}$$

For parallel-group trials, the pooled SD was calculated as:

$$SD_{pooled}=\sqrt{\frac{\left(n_{CHOE}-1\right)SD_{CHOE}^{2}+\left(n_{CON}-1\right)SD_{CON}^{2}}{n_{CHOE}+n_{CON}-2}}$$

For crossover trials, the effective degrees of freedom, exact Hedges correction, and corrected standardized mean difference were calculated as:

$$\nu =\frac{2(n-1)}{1+{r_{\mathrm{pair}}}^{2}}$$

$$J(\nu )=\frac{\Gamma (\frac{\nu }{2})}{\sqrt{\frac{\nu }{2}}\Gamma (\frac{\nu -1}{2})}$$

$$g=J(\nu )\cdot \frac{M_{\mathrm{CHO}\text{-}E}-M_{\mathrm{CON}}}{{SD}_{\mathrm{av}}}$$

For parallel-group trials, Hedges’ g was calculated as:

$$g=\frac{M_{CHOE}-M_{CON}}{SD_{pooled}}\times \left(1-\frac{3}{4\left(n_{CHOE}+n_{CON}-2\right)-1}\right)$$

For within-subject crossover trials standardized with SD_av, the sampling variance and standard error of Hedges’ g were calculated as:

$$v_{g}=\frac{2(1-r_{\mathrm{pair}})}{n}+\frac{g^{2}(1+{r_{\mathrm{pair}}}^{2})}{4n}$$

$${SE}_{g}=\sqrt{v_{g}}$$

For parallel-group trials, the SE of Hedges’ g was calculated as:

$$SE_{g}=\sqrt{\frac{n_{CHOE}+n_{CON}}{n_{CHOE}\times n_{CON}}+\frac{g^{2}}{2\left(n_{CHOE}+n_{CON}\right)}}$$

For both crossover and parallel-group estimates, the sampling variance was the squared standard error:

$$v_{g}={{SE}_{g}}^{2}$$

#### 2.6.2. Meta-Analysis and Heterogeneity

All data manipulation, graphical visualization, and statistical analyses were performed using R software (version 4.5.1; R Foundation for Statistical Computing). Data wrangling and customized plotting were facilitated by the tidyverse package suite, particularly dplyr and ggplot2 [31]. To account for statistical dependency arising from multiple eligible outcomes, contrasts, or intervention arms reported within the same study, three-level random-effects meta-analytic models were fitted using the rma.mv function in the metafor package. Hedges’ g was used as the observed effect size, and the corresponding sampling variance–covariance matrix was specified as the known level-1 sampling error structure. Effect sizes were nested within studies using a random-effects structure equivalent to random = ~1|study/effect_size, allowing the model to partition the total variability into sampling variance, within-study heterogeneity among effect sizes, and between-study heterogeneity across studies [32].

Model parameters were estimated using the restricted maximum likelihood (REML) method. The robustness of the model estimates was evaluated through methodological comparisons with maximum likelihood (ML) estimation, as well as through subsequent sensitivity analyses [33,34]. Tests of individual coefficients and their corresponding 95% confidence intervals (CIs) were calculated using the t-distribution [35]. Furthermore, 95% prediction intervals (PIs) were computed to estimate the range within which the true effects of comparable future studies are expected to fall [36,37].

Heterogeneity was assessed by estimating the variance components at both the within-study and between-study levels. Specifically, the level-2 variance component represented heterogeneity among multiple effect sizes within the same study, whereas the level-3 variance component represented heterogeneity between studies. Multilevel *I*^2^ statistics were calculated to quantify the proportion of total variability attributable to true heterogeneity rather than sampling error, including both total and level-specific *I*^2^ estimates. These values were interpreted as low (0–25%), moderate (25–49%), substantial (50–74%), or considerable (≥75%) [38]. Exploratory observed-power plots generated during the original analysis are retained only in Supplementary Material S8 for transparency [39]. Because observed power is a direct function of the estimated effect and its standard error, these plots were not used to support inference, robustness, or certainty; interpretation relied on effect estimates, CIs, PIs, heterogeneity, and GRADE.

#### 2.6.3. Moderator and Subgroup Analyses

Four outcome-domain categories were used in the originally submitted analysis according to the physiological construct represented by each performance outcome: aerobic endurance (e.g., time trials and time to exhaustion), anaerobic endurance (e.g., repeated high-intensity bouts), explosive power (e.g., jumps and short sprints), and strength (e.g., maximal voluntary contraction). During revision, the reporting hierarchy was amended to give these construct-specific estimates—particularly aerobic endurance—primary interpretive priority, while retaining the cross-domain omnibus estimate as secondary context. This reporting amendment did not alter the eligibility criteria or the outcome-domain classifications used in the original submission. Subgroup estimates were interpreted cautiously when a category contained fewer than five studies [40].

As a post hoc analysis, aerobic outcomes were further separated using a strict protocol-based definition. The time-trial/fixed-work-completion category comprised tasks in which a fixed amount of work or distance was completed as quickly as possible; the explicit time-to-exhaustion category required an outcome reported as time to exhaustion. Three aerobic outcomes did not satisfy either strict definition: work completed during a fixed 30 min test, distance accumulated in a protocol with multiple possible stopping criteria, and cumulative time in a termination-limited loaded-marching protocol. These outcomes were retained in the full aerobic domain model but excluded from the strict comparison. A broader sensitivity classification assigned the fixed-duration work test to performance and the two termination-limited tests to exercise capacity. Separate three-level REML models were fitted for each category, and a mixed-effects meta-regression tested the between-category difference.

Furthermore, exploratory meta-regression analyses assessed ambient temperature (°C) and reported environmental-exposure duration as continuous moderators. Both variables were mean-centered before model fitting, with duration rescaled to 10 min units. Meta-regression was applied only when at least ten independent studies were available [25], using complete-case analyses for the respective moderator variables.

All moderator analyses were executed using three-level, mixed-effects models with restricted maximum likelihood (REML) estimation via the metafor package (rma.mv function) in R [29]. To account for statistical dependence, effect sizes were nested within studies via the variance–covariance matrix. Statistical inference relied on t-distribution tests with containment-based degrees of freedom. Due to sample size constraints, exclusively linear models were fitted. Given the exploratory nature of these analyses and the absence of multiplicity adjustments, interpretations prioritized estimate magnitudes, confidence interval overlap, and overall robustness over isolated statistical significance. Visualizations were generated using ggplot2 [31], with meta-regression bubble sizes proportional to effect-size precision.

### 2.7. Publication Bias and Sensitivity Analyses

Potential small-study effects were assessed visually with contour-enhanced funnel plots and, where at least ten studies were available, with a cluster-aware Egger-type meta-regression in which the sampling standard error of each effect estimate was entered as the moderator. The model retained the full sampling variance–covariance matrix and the three-level structure [41,42,43]. For the descriptive study-level funnel plot, the corrected effect estimates from study i were combined as θ_i_ = (1′V_i_^−1^y_i_)/(1′V_i_^−1^1), with variance (1′V_i_^−1^1)^−1^, where V_i_ is the study-specific block of the corrected sampling variance–covariance matrix. Because conventional trim-and-fill assumes independent effects [44], and its result would not resolve publication bias in this dependent multilevel dataset, it was not used for inferential adjustment. Asymmetry findings were interpreted as exploratory evidence about small-study effects, not as direct proof of publication bias or its absence.

Sensitivity analyses were conducted within the three-level meta-analytic framework to evaluate robustness. First, a leave-one-study-out influence analysis iteratively omitted individual studies. Second, the complete SMCRP calculations for crossover trials were repeated using alternative within-participant paired-condition correlations (r_pair = 0.20 and r_pair = 0.80) instead of the primary r_pair = 0.50, while the separate within-study sampling-error dependence parameter remained fixed at ρ = 0.50. Third, post hoc ambient-temperature threshold analyses refitted the same corrected three-level REML model after excluding studies conducted at 22.0–25.0 °C (>25 °C subset) and after applying a stricter ≥28 °C threshold. The latter is consistent with a previously published operational criterion for exercise in hot environments [45]; neither cutoff was interpreted as a universal physiological heat-stress threshold.

### 2.8. Certainty of the Evidence

The Grading of Recommendations Assessment, Development, and Evaluation (GRADE) framework was applied separately to each synthesis using the five domains of risk of bias, inconsistency, indirectness, imprecision, and publication bias [46]. The evidence base is reported as unique studies, effect sizes (K), and unique participants (N); participants in crossover trials and participants contributing multiple effects within one outcome were counted once. Risk-of-bias downgrading was based on the revised RoB 2 judgments among studies contributing to that outcome. Imprecision considered the 95% CI and plausible effect range together with total N; N < 400 was used as a working heuristic for SMD evidence rather than as an isolated absolute rule. Non-significant or underpowered asymmetry tests were not interpreted as evidence that publication bias was absent. Outcome-specific reasons for every downgrade are provided in Supplementary Material S4. Assessments were performed by X.W. and independently verified by T.L.

### 2.9. Direction Standardization

To ensure interpretative consistency across outcomes, all effect sizes were aligned to a benefit-oriented scale. For measures where lower values indicate enhanced performance (e.g., sprint time, total completion time), Hedges’ g estimates were multiplied by −1. This directional adjustment strictly preserved the absolute magnitudes and sampling variances. Consequently, a positive effect size (g>0) uniformly denotes an ergogenic benefit favoring the CHO-E intervention.

## 3. Results

### 3.1. Study Selection

A total of 135,191 records were identified from PubMed (n = 27,741), Embase (n = 30,240), Web of Science (n = 42,394), CENTRAL (n = 4280), and Scopus (n = 30,536). After 44,092 duplicates were removed, 91,099 deduplicated records were loaded into ASReview for prioritization. Human reviewers labeled 611 records (135 relevant and 476 irrelevant, including the 26 priors), and 90,488 records remained unlabeled when the prespecified threshold of 300 consecutive irrelevant records was met. The 135 relevant records were exported to Zotero. Fifty-two records were removed during Zotero verification (seven residual duplicates and 45 title/abstract exclusions), leaving 83 reports sought for retrieval. Full texts could not be obtained for 52 reports, and 31 reports were assessed for eligibility. Ten reports were excluded because of non-acute supplementation (n = 1), unclear test indicators (n = 1), unpublished gray literature (n = 1), ineligible control supplementation (n = 1), absence of performance-related outcomes (n = 1), inappropriate participant age (n = 1), no supplementation in the control group (n = 3), or insufficient quantitative ambient-temperature reporting to verify the ≥22 °C criterion (n = 1; Hsu et al. [47]). Finally, 21 studies were included in the systematic review and meta-analysis [10,11,48,49,50,51,52,53,54,55,56,57,58,59,60,61,62,63,64,65,66]. Of these, one was a cluster-assigned parallel trial and the remaining 20 used crossover designs (Figure 1).

### 3.2. Characteristics of the Studies

The 21 included studies involved 316 participants (282 men and 34 women), with sample sizes ranging from seven to 44. Participants represented a broad performance continuum, from recreationally active individuals to trained, competitive, and occupationally active performers. Twenty studies used crossover designs, and one was a cluster-assigned parallel trial. Reported mean ages ranged from 15.6 to 32.3 years, and ambient temperatures ranged from 22.0 °C to 36.0 °C.

Exercise protocols included running, cycling, intermittent shuttle-running, repeated-sprint tests, Wingate cycling, CMJ, and MVC/MVIC assessments. CHO-E supplementation varied in carbohydrate concentration, fluid volume, and timing, including pre-exercise, during-exercise, recovery-period, and combined supplementation strategies. Reported outcomes included time to exhaustion, time-trial performance, total work or distance, repeated-sprint performance, mean power, CMJ, and MVC/MVIC. For more details, please refer to Supplementary Material S3.

### 3.3. Risk of Bias

Following report-based reassessment, 19 of 21 studies (90.5%) raised some concerns overall and two (9.5%) were judged at high risk; none was low risk overall (Figure 2). All 21 studies raised some concerns in the randomization domain because allocation concealment before enrolment and assignment was not adequately established in the available reports. Bergen et al. reported random order assignment by an independent research assistant who retained the drink code, but did not establish that enrolling or assigning personnel could not foresee the sequence before enrolment and assignment. Among 20 crossover studies, period/carryover risk was low in 10 and raised some concerns in 10; it was not applicable to the cluster-assigned parallel trial. This judgment depended on sequence balance, period-effect handling, and the interval before second-period assessment, not on crossover design itself. The deviations, missing-outcome, and outcome-measurement domains were generally low risk. Exceptions were Criswell et al. (high risk in deviations/analysis because treatment was assigned by team but participant-level analyses did not account for clustering) and Millard-Stafford et al. (1992) [64] (high risk for missing outcome data because two of eight placebo participants did not complete 40 km and their completion times were extrapolated). All studies raised some concerns in selection of the reported result because a pre-unblinding analysis plan could not be verified. Full signaling-question responses and rationales are reported in Supplementary Material S9.

In the independently re-audited descriptive PEDro checklist, study totals ranged from two to six of 10 (mean 4.9; median five). These values summarize explicitly reported design and reporting features only; they were not used to revise the RoB 2 or GRADE assessments. Item-level judgments and definitions are provided in Supplementary Material S12.

In the cluster-aware Egger-type meta-regression for the secondary overall synthesis, sampling standard error was entered as the moderator. The estimated slope was 2.67 (95% CI: −0.25 to 5.59; *p* = 0.071), providing inconclusive evidence about small-study effects. The study-level generalized-least-squares funnel plot was treated as descriptive, and no trim-and-fill inference was used (Supplementary Material S5). Exploratory post hoc power visualizations are retained only in Supplementary Material S8 and were not used for inference or to strengthen the conclusions.

### 3.4. Secondary Overall (Omnibus) Synthesis

In the secondary cross-domain omnibus synthesis estimated using a three-level random-effects model with restricted maximum likelihood, CHO-E supplementation was associated with a positive average difference in direct exercise performance compared with control conditions. Across 32 effect sizes from 21 studies and 316 participants, the pooled estimate favored CHO-E supplementation (Hedges’ g = 0.37, 95% CI: 0.21 to 0.53, *p* < 0.001), corresponding to a small average effect. This estimate summarizes the eligible direct-performance evidence on a common scale and should not be interpreted as demonstrating interchangeability among the contributing test types.

The prediction interval was wider and crossed the null (PI: −0.21 to 0.96), indicating that effects in future comparable settings may range from potentially unfavorable to clearly beneficial. Moderate heterogeneity was observed (*I*^2^ = 46.7%). Variance decomposition suggested that heterogeneity was concentrated at the within-study effect-size level (level 2 = 46.7%), with no detectable between-study heterogeneity in this model (level 3 ≈ 0%) [32,33,34]. After outcome-specific reassessment of risk of bias and imprecision, the certainty of evidence was rated as low according to GRADE (Figure 3; Supplementary Material S4).

### 3.5. Primary Outcome-Domain and Exploratory Moderator Analyses

#### 3.5.1. Primary Outcome-Domain Analyses

The primary outcome-domain analyses revealed a clear outcome-dependent pattern. CHO-E supplementation was associated with a positive average effect in aerobic endurance (15 studies, 21 effect sizes: Hedges’ g = 0.45 [95% CI, 0.26 to 0.64], *p* < 0.001). Heterogeneity was concentrated at the study level (*I*^2^-L3 = 42.9%; *I*^2^-L2 = 0.0%). However, the prediction interval crossed zero (PI: −0.16 to 1.02), and GRADE certainty was low.

Conversely, estimates were statistically inconclusive for anaerobic endurance (g = 0.19 [95% CI, −0.29 to 0.66], *p* = 0.417), explosive power (g = 0.18 [95% CI, −0.20 to 0.55], *p* = 0.339; *I*^2^-L2 = 77.1%), and strength (g = 0.38 [95% CI, −0.15 to 0.92], *p* = 0.155). These domains contained only two to four studies and three to five effect sizes, total N ranged from 21 to 89, and the confidence and prediction intervals were wide. GRADE certainty was therefore very low for all three non-aerobic domains (Figure 4; Supplementary Material S4).

#### 3.5.2. Post Hoc Time-Trial Versus Time-to-Exhaustion Analyses

The strict post hoc analysis included 12 time-trial/fixed-work-completion effects from seven studies and six explicit time-to-exhaustion effects from five studies. The average effects favored CHO-E in both categories: time-trial/fixed-work completion, g = 0.41 (95% CI, 0.12 to 0.69; *p* = 0.014; 95% PI, −0.11 to 0.92; *I*^2^ = 26.21%); and explicit time to exhaustion, g = 0.66 (95% CI, 0.06 to 1.26; *p* = 0.038; 95% PI, −0.57 to 1.89; *I*^2^ = 59.89%). Both prediction intervals crossed zero, and the time-to-exhaustion estimate was less precise and more heterogeneous.

The mixed-effects comparison did not identify a clear difference between the strict test categories (β = 0.25, 95% CI, −0.26 to 0.76; *p* = 0.303). In the broader sensitivity classification, the pooled effects were g = 0.40 (95% CI, 0.16 to 0.64) for performance-type tests and g = 0.49 (95% CI, 0.01 to 0.97) for capacity-to-termination tests, again without a clear between-category difference (β = 0.09; *p* = 0.668). These analyses are reported in Supplementary Material S10 and should be considered exploratory because they were added post hoc and include few studies.

#### 3.5.3. Meta-Regression by Ambient Temperature and Reported Exposure Duration

Three-level meta-regression analyses revealed that neither ambient temperature nor exposure duration significantly moderated the CHO-E-associated performance difference. For ambient temperature (21 studies, 32 effect sizes), the slope was small and non-significant (β = 0.013 per °C [95% CI, −0.026 to 0.052], *p* = 0.485). Exposure duration (13 studies, 18 effect sizes) was likewise non-significant (β = 0.013 per 10 min [95% CI, −0.035 to 0.061], *p* = 0.565).

In both models, residual heterogeneity was confined to the effect-size level (temperature: *I*^2^-L2 = 47.2%, *I*^2^-L3 ≈ 0%; duration: *I*^2^-L2 = 59.6%, *I*^2^-L3 ≈ 0%). Linear moderation was not confirmed within the observed ranges, and precision decreased at the extremes of the moderator distributions (Figure 5).

### 3.6. Sensitivity Analyses

To assess robustness to the paired-condition correlation assumption, the complete crossover calculations were repeated using r_pair = 0.20, 0.50, and 0.80 while the separate within-study dependence parameter was fixed at ρ = 0.50. The pooled estimates were stable (Hedges’ g range: 0.36–0.37; all *p* < 0.001), although higher r_pair values produced wider prediction intervals and greater estimated heterogeneity. REML and ML yielded essentially identical pooled estimates (g = 0.37 for both) (Supplementary Material S6).

Leave-one-study-out analyses confirmed that the effects for overall performance (g range: 0.34–0.40) and aerobic endurance (g range: 0.41–0.49) were not driven by any single study (Supplementary Material S7). Omission-based results for the sparsely represented non-aerobic domains were considered unstable and were therefore interpreted descriptively rather than as confirmatory evidence.

Post hoc ambient-temperature threshold analyses did not indicate that the secondary overall mean estimate depended on trials conducted at 22–25 °C. After those trials were excluded (>25 °C only), 18 studies, 27 effect sizes, and 273 participants yielded g = 0.41 (95% CI: 0.23 to 0.59; *p* < 0.001; 95% PI: −0.25 to 1.07; *I*^2^ = 51.2%). At a stricter threshold of ≥28 °C, 13 studies, 19 effect sizes, and 188 participants yielded g = 0.36 (95% CI: 0.11 to 0.61; *p* = 0.009; 95% PI: −0.47 to 1.19; *I*^2^ = 58.3%). The mean direction remained positive, but both prediction intervals included the null and the higher restriction reduced precision (Supplementary Material S11).

## 4. Discussion

### 4.1. Principal Findings and Outcome-Specific Interpretation

This systematic review and three-level meta-analysis evaluated acute CHO-E supplementation during exercise conducted at reported ambient temperatures of ≥22 °C, the operational eligibility range used in the review spanning moderate or mildly warm to hot conditions. Giving primary interpretive priority to analyses based on the four outcome-domain categories, the clearest finding was a small positive average estimate for aerobic endurance, although its prediction interval crossed zero and the certainty of evidence was low. The secondary cross-domain omnibus synthesis also yielded a small positive average estimate, but it should be read as a broad summary of eligible direct-performance outcomes rather than as a task-specific or heat-protective effect.

Use of a common standardized scale permits outcomes measured in different units to be summarized, and the three-level model appropriately represents dependence among multiple effects from the same study. Neither feature, however, makes time trials, time to exhaustion, sprint tests, jumps, or maximal contractions physiologically or psychometrically interchangeable. The overall estimate therefore describes an average across a heterogeneous performance evidence base; domain- and protocol-specific estimates provide the more relevant basis for practical interpretation.

### 4.2. Outcome-Specific Findings Within the Specified Ambient-Temperature Range

Within the specified ambient-temperature range, the clearest average performance difference was observed in aerobic endurance. This pattern is compatible with the substrate and fluid demands of prolonged exercise [7], but the included trials compared CHO-E with control within the same environmental condition. They did not compare hot with thermoneutral exercise under the same supplementation condition and therefore do not estimate whether CHO-E modifies or offsets the causal effect of heat itself.

Within aerobic endurance, the strict post hoc analyses yielded positive average estimates for both time-trial/fixed-work-completion and explicit time-to-exhaustion tests, but the between-type difference was inconclusive. The time-to-exhaustion estimate had greater heterogeneity and a substantially wider prediction interval, consistent with concerns about the variability and reliability of these protocols. Three aerobic outcomes that did not map cleanly to either strict definition were retained in the full aerobic model but excluded from the strict comparison. Accordingly, the split analyses do not support treating the two protocols as equivalent; rather, they suggest positive average effects in both small evidence subsets while leaving considerable uncertainty about future settings.

Conversely, evidence for non-aerobic domains (anaerobic endurance, explosive power, and strength) was statistically inconclusive. This uncertainty reflects sparse evidence and wide intervals rather than evidence that supplementation is ineffective. Brief, explosive tasks depend more heavily on neuromuscular recruitment and phosphagen availability, which may be less sensitive to acute exogenous substrate delivery than prolonged aerobic efforts [67]; however, the synthesis evaluated performance outcomes rather than physiological mediators directly.

### 4.3. Environmental Moderators: Complex Interactions Beyond Linear Models

Exploratory meta-regression did not identify ambient temperature or exposure duration as significant linear moderators. Post hoc threshold sensitivity analyses also retained positive mean estimates after excluding trials at 22–25 °C and after restricting the evidence base to ≥28 °C. However, the prediction intervals crossed zero in both threshold analyses and precision decreased as the evidence base became smaller. These findings support stability of the mean direction, not uniform benefit across settings and not a heat-specific protective interaction.

Thermal strain is a multifactorial construct. Dry-bulb ambient temperature alone provides an incomplete description of environmental severity without relative humidity, radiant heat, airflow, clothing, metabolic heat production, exposure duration, and acclimation status [1,68]. Inconsistent reporting of these factors across primary studies limits interpretation of the continuous and threshold analyses. The widening of confidence bands at exposure extremes also highlights reduced precision where data are sparse.

### 4.4. Quality of Evidence and Robustness of Findings

The robustness of the numerical estimates was evaluated across estimation methods, paired-condition correlation assumptions, leave-one-study-out analyses, and ambient-temperature thresholds. Overall and aerobic estimates were not driven by a single study, and the secondary overall mean remained positive under the two higher-temperature restrictions. Nevertheless, prediction intervals crossed zero and the certainty of evidence was low or very low, so stability of an average estimate should not be equated with certainty of benefit in every future setting.

Methodologically, the revised RoB 2 assessment identified 19 studies with some concerns and two at high risk overall. The principal recurring limitations were unreported allocation concealment and the absence of a verifiable analysis plan finalized before unblinding; two study-specific high-risk judgments concerned ignored team-level clustering and outcome-dependent missing performance data. Period and carryover effects were evaluated using reported sequence balance, period-effect analyses, and the time between outcome assessments; no judgment was inferred from crossover design alone. These judgments concern susceptibility to bias and do not address research misconduct. The revised GRADE certainty was low for overall exercise performance and aerobic endurance and very low for anaerobic endurance, explosive power, and strength, driven primarily by risk of bias and imprecision and, for explosive power, inconsistency.

### 4.5. Strengths of the Review

This review used a three-level random-effects model to represent dependence among multiple effects from the same study [33] and applied design-specific effect-size calculations for crossover and cluster-assigned parallel evidence. Eligibility excluded multi-ingredient formulations containing independent ergogenic aids, thereby focusing the synthesis on CHO-E versus water or non-caloric placebo. The environmental boundary and its limitations are now reported explicitly, and two higher-temperature restrictions were examined post hoc. Because the eligible comparisons were CHO-E formulations versus water or non-caloric controls, they do not isolate the independent contributions of carbohydrate, electrolytes, fluid volume, palatability, or their interaction.

A further strength is the combined reporting of confidence intervals, prediction intervals, outcome-specific GRADE assessments, and influence analyses. Together, these components distinguish the average estimate from uncertainty about future settings and make the sparse evidence in non-aerobic domains explicit.

### 4.6. Limitations

Despite these strengths, several limitations warrant consideration. First, the available evidence base remains constrained by small sample sizes and a substantial sex disparity (282 men vs. 34 women), limiting generalizability to female athletes and preventing adequately powered sex-specific or non-aerobic subgroup analyses. The small female sample also precluded a reliable sex subgroup analysis, so the pooled estimates should not be assumed to apply equally to female athletes. Sex-related differences in substrate utilization, thermoregulatory responses, sweating and electrolyte losses, and hormonal milieu could plausibly modify responses, but the available dataset cannot test those mechanisms. Generalization to female athletes should therefore be cautious.

Second, the included literature exhibited substantial heterogeneity in exercise-test constructs, protocols, supplementation strategies, and measurement paradigms. Although Hedges’ g and benefit-oriented direction alignment enabled an omnibus synthesis, standardization does not establish physiological or practical interchangeability; the overall pooled estimate should therefore not be translated directly to a specific task. The time-trial versus time-to-exhaustion analyses were added post hoc, included only seven and five studies in the strict categories, and excluded three aerobic outcomes that did not meet either strict definition. These category-specific results are therefore exploratory. In addition, the heavy reliance on crossover designs required imputation of paired-condition correlations, although sensitivity analyses indicated that the pooled estimates were stable across the examined values. Variation in carbohydrate concentration, electrolyte composition, fluid volume, supplementation timing, and comparator formulation further limits prescription-level conclusions; these factors could not be disentangled reliably with the available reporting and number of independent studies.

Third, full texts could not be obtained for 52 of the 83 reports sought for retrieval, and 90,488 ASReview records remained unlabeled when the prespecified stopping criterion was met. Consequently, some eligible studies may have remained unidentified, potentially limiting the completeness of the evidence base.

Finally, ≥22 °C is an operational eligibility boundary rather than a universal physiological heat-stress threshold. The primary studies inconsistently reported humidity, radiant heat, airflow, clothing, exposure duration, and acclimation status; One otherwise relevant report was excluded because neither quantitative ambient temperature nor WBGT was reported in sufficient detail to verify eligibility [47]. Although the mean estimate remained positive in the >25 °C and ≥28 °C sensitivity subsets, both prediction intervals crossed zero and the smaller evidence bases reduced precision. Safety and adverse events were not systematically assessed; therefore, this review cannot support a conclusion regarding the safety or ‘low-risk’ status of acute CHO-E supplementation. The evidence base also includes heterogeneous publication formats; although only reports with extractable randomized performance data were retained, publication format may affect reporting completeness.

### 4.7. Practical Implications and Translational Perspectives

From a translational perspective, the small average estimate may be relevant in some endurance settings, but low certainty and prediction intervals crossing zero preclude a universal claim of benefit. Acute CHO-E may be considered as one practical fueling and hydration option for aerobic endurance performed within the studied temperature range; the present evidence does not establish a heat-specific protective effect.

Practical recommendations should remain task- and context-specific. Current evidence is inconclusive for short-duration, explosive, anaerobic, and strength outcomes. CHO-E should complement, not replace, established heat-management measures such as acclimation, pacing, cooling, and individualized hydration planning [69]. Future trials should recruit more women, report multidimensional environmental exposure including WBGT components, and directly test supplementation-by-environment interactions when the aim is to determine heat-specific protection.

## 5. Conclusions

Among randomized trials conducted at reported ambient temperatures of ≥22 °C, acute CHO-E supplementation was associated with a small positive average difference in direct exercise performance, with the clearest estimate in aerobic endurance. Evidence for anaerobic endurance, explosive power, and strength remained inconclusive because few studies and participants contributed to these domains.

These treatment comparisons were made within the same environmental condition and therefore do not establish that CHO-E offsets heat-induced performance decrements. Positive mean estimates were retained after excluding 22–25 °C studies and at ≥28 °C, but prediction intervals crossed zero and precision decreased. Given low or very low certainty, future research should improve sex representation, environmental reporting, task-specific replication, and direct hot-versus-thermoneutral interaction testing. Because the prediction intervals cross the null, the effect in some future comparable settings may be absent or unfavorable.

## Figures and Tables

**Figure 1:**
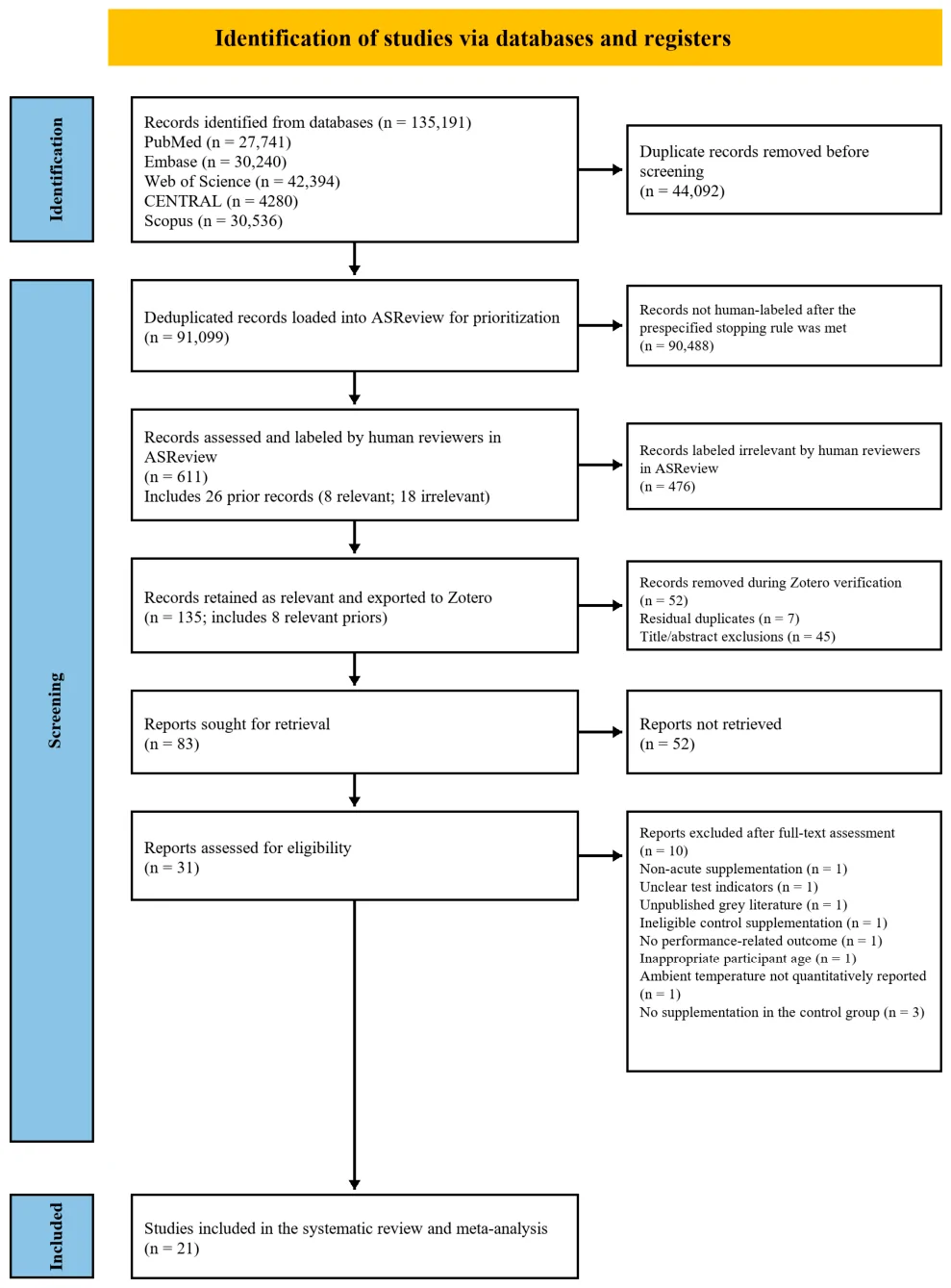
PRISMA flow diagram of the study selection process for the systematic review and meta-analysis.

**Figure 2:**
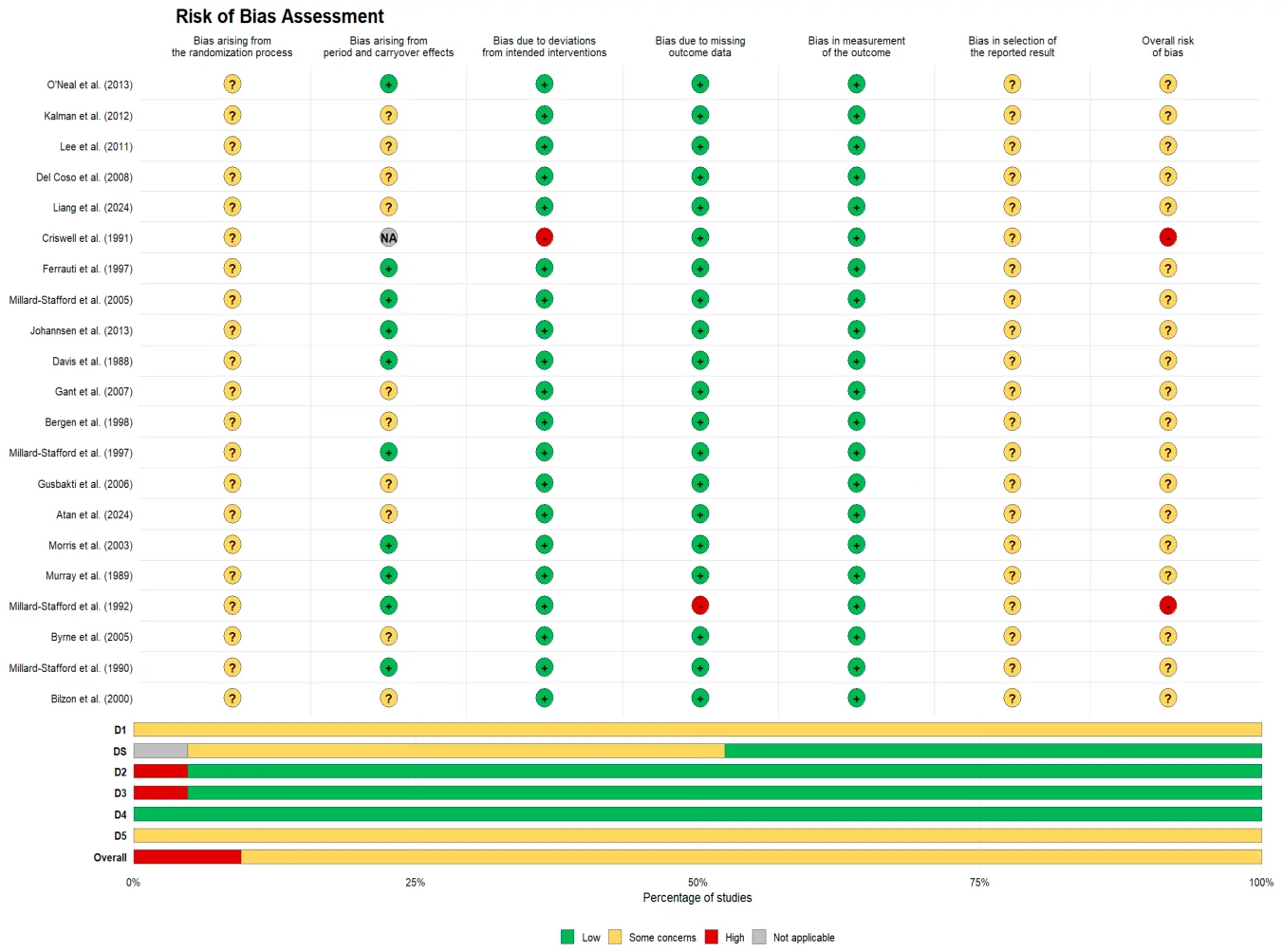
Revised study-level RoB 2 summary for eligible direct-performance results. The overall study judgment is the least favorable across assessed domains; (+) low risk of bias; (?) some concerns; (−) high risk of bias; (NA) not applicable. Detailed signaling-question responses and rationales are provided in Supplementary Material S9. The studies presented in this figure correspond to references [10,11,48,49,50,51,52,53,54,55,56,57,58,59,60,61,62,63,64,65,66].

**Figure 3:**
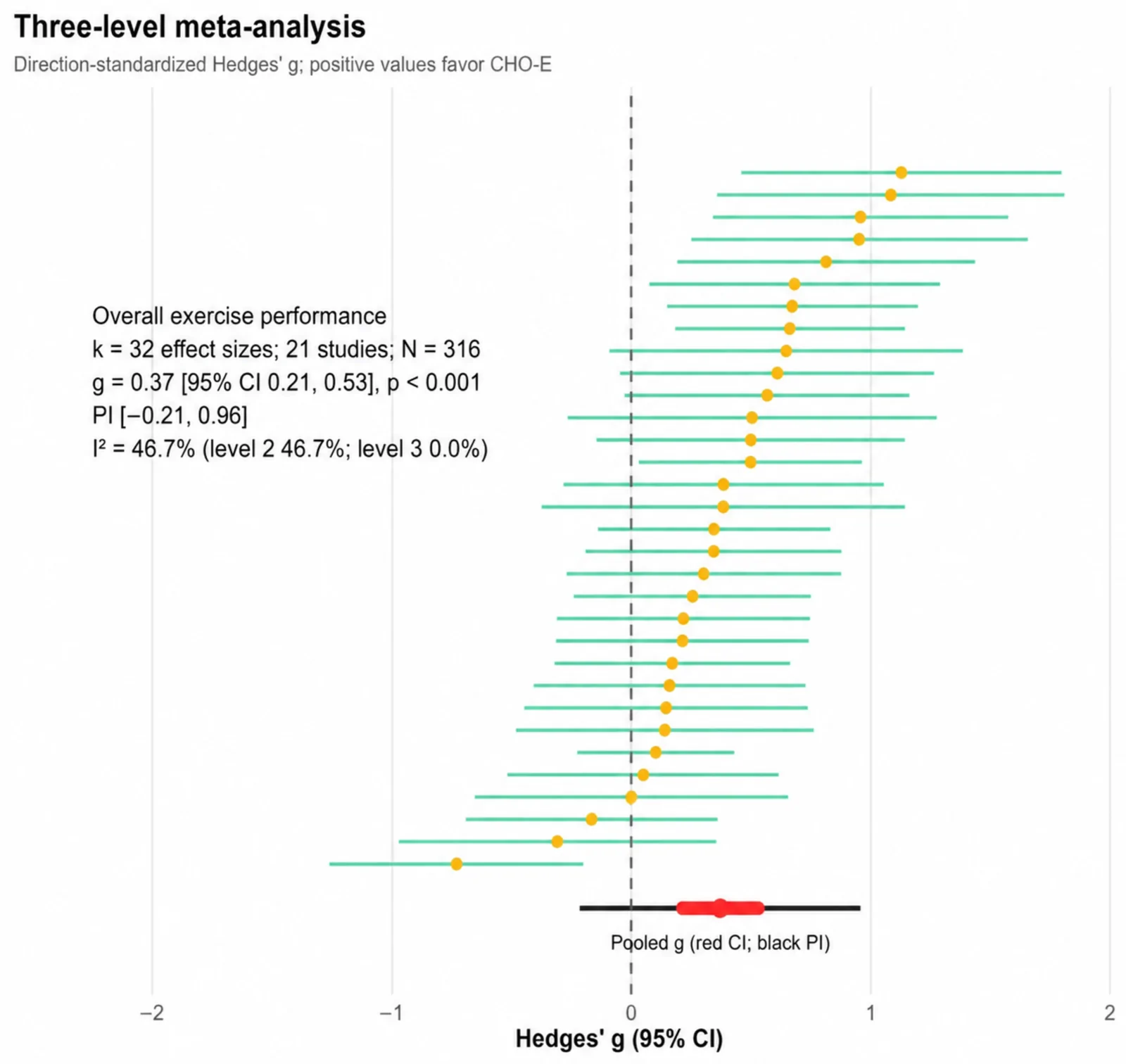
Pooled effect of acute carbohydrate–electrolyte supplementation on overall exercise performance after correction of the crossover effect-size calculations. K, number of effect sizes; Hedges’ g, standardized mean difference corrected for small-sample bias; CI, confidence interval; PI, prediction interval; *I*^2^, proportion of variability attributable to heterogeneity.

**Figure 4:**
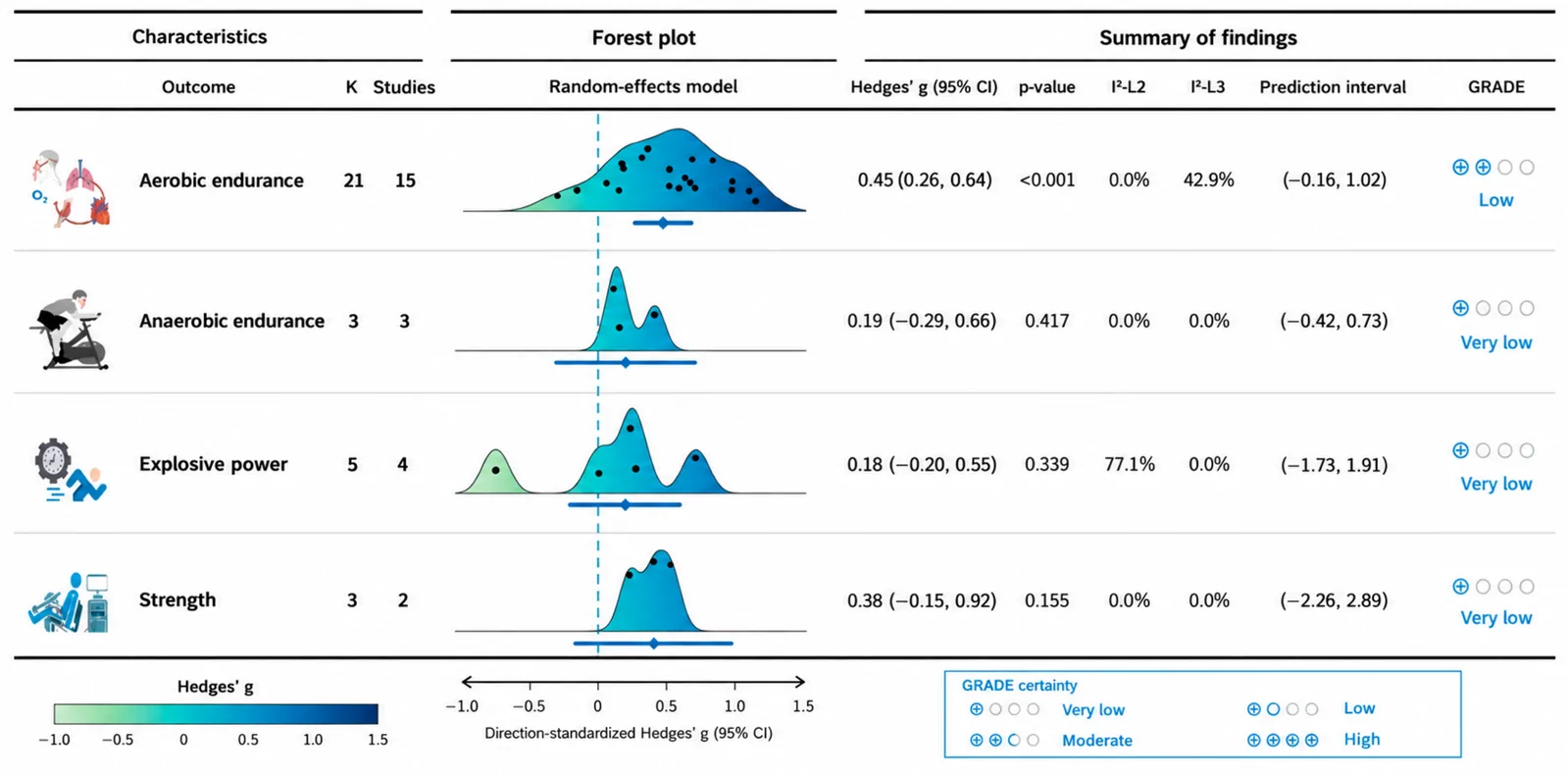
Domain-specific analysis of acute CHO-E supplementation and exercise performance after correction of the crossover effect-size calculations and exclusion of the report without verifiable quantitative ambient temperature. Black points show observed effect sizes; their vertical positions are used only to display the observations and have no statistical meaning. Mountain contours are kernel-density displays normalized separately within each outcome domain, so peak heights should not be compared across domains. Horizontal intervals show the model estimates. CI, confidence interval; Hedges’ g, standardized effect size corrected for small-sample bias; *I*^2^-L2 and *I*^2^-L3, heterogeneity at the within-study/effect-size and between-study levels, respectively; K, number of effect sizes; CHO-E, carbohydrate–electrolyte.

**Figure 5:**
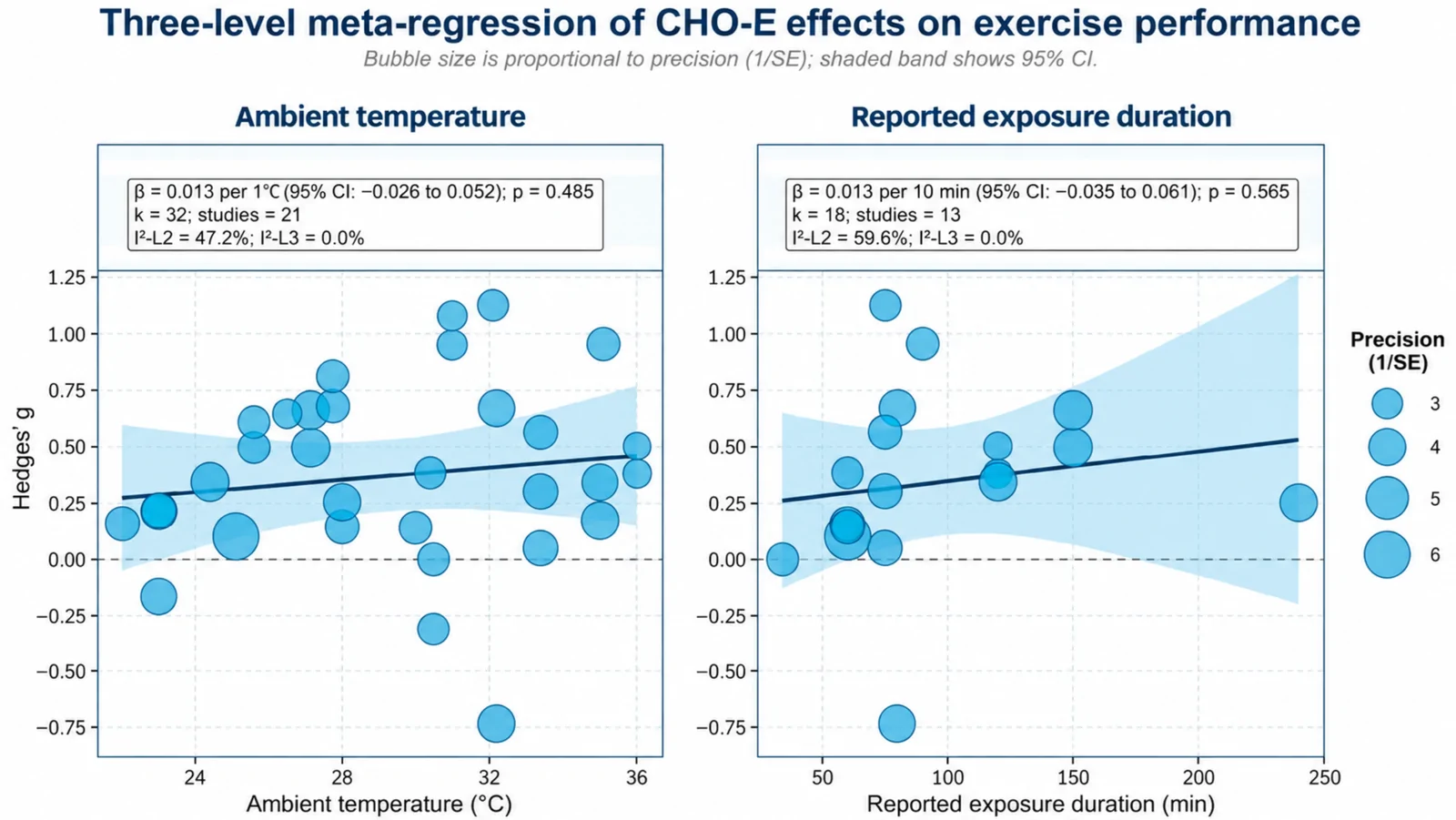
Three-level meta-regression of CHO-E-associated performance differences by ambient temperature and reported environmental-exposure duration. Bubble size is proportional to precision (1/SE); solid lines are fitted slopes and shaded areas are 95% confidence intervals. Positive effect sizes favor CHO-E supplementation.

## Data Availability

All data analyzed in this study were obtained from previously published studies, which are cited in the manuscript. No new data were generated for this study.

## Supplementary Materials

The following supporting information can be downloaded at: https://www.mdpi.com/article/10.3390/nu18193200/s1. Supplementary Material S1: Search strategy. Supplementary Material S2: PRISMA 2020 Checklist. Supplementary Material S3: Study characteristics. Supplementary Material S4: GRADE Assessment. Supplementary Material S5: Funnel plot. Supplementary Material S6: Summary of Sensitivity Analysis Results. Supplementary Material S7: A Sensitivity Analysis Based on Level-3 Leave one-out. Supplementary Material S8: Power Visualization. Supplementary Material S9: Study-level RoB 2 Signalling-Question Responses and Rationales. Supplementary Material S10: Time-Trial and Time-to-Exhaustion Outcome-Type Analyses. Supplementary Material S11: Ambient-Temperature Threshold Sensitivity Analyses. Supplementary Material S12: Conservative PEDro Checklist Audit; Descriptive Only. Supplementary Material S13: Data-Source Provenance Audit.

## Abbreviations

The following abbreviations are used in this manuscript:

CHO-E	carbohydrate–electrolyte
CI	confidence interval
PI	prediction interval
RCT	randomized controlled trial
RoB 2	revised Cochrane risk-of-bias tool for randomized trials
GRADE	Grading of Recommendations Assessment, Development and Evaluation
REML	restricted maximum likelihood
ML	maximum likelihood
SMD	standardized mean difference
CMJ	countermovement jump
MVC	maximal voluntary contraction
MVIC	maximal voluntary isometric contraction.
