# Supplementary material for: Effects of Acute Carbohydrate–Electrolyte Supplementation on Exercise Performance in Moderate-to-Hot Ambient Conditions: A Systematic Review and Meta-Analysis
Source: Nutrients. 2026 Sep 28;18(19):3200. doi: 10.3390/nu18193200 (PMC13666840; doi:10.3390/nu18193200)
Supplement: Supplementary file 1 [file nutrients-18-03200-s001.zip › nutrients-4463583-supplementary.pdf]

Number	Material
1	Supplementary Material S1 (Search strategy)
2	Supplementary Material S2 (PRISMA 2020 Checklist)
3	Supplementary Material S3 (Study characteristics)
4	Supplementary Material S4 (GRADE Assessment)
5	Supplementary Material S5 (Funnel plot)
6	Supplementary Material S6<br>(Summary of Sensitivity Analysis Results)
7	Supplementary Material S7<br>(A Sensitivity Analysis Based on Level-3 Leave one-out)
8	Supplementary Material S8 (Power Visualization)
9	Supplementary Material S9 (Study-level RoB 2 Signalling-Question Responses and Rationales)
10	Supplementary Material S10 (Time-Trial and Time-to-Exhaustion Outcome-Type Analyses)
11	Supplementary Material S11 (Ambient-Temperature Threshold Sensitivity Analyses)
12	Supplementary Material S12 (Conservative PEDro Checklist Audit; Descriptive Only)
13	Supplementary Material S13 (Data-Source Provenance Audit)

Supplementary Material S1 (Search strategy)

Database, platform, and search date	Retrospectively adapted database-specific reporting syntax	Records retrieved
**PubMed**<br>Platform: NCBI PubMed<br>Date searched: 27 May 2026	(((("Carbohydrates"[MeSH Terms] OR "Glucose"[MeSH Terms] OR "Fructose"[MeSH Terms] OR "Sucrose"[MeSH Terms] OR "Maltose"[MeSH Terms] OR "Dextrins"[MeSH Terms] OR carbohydrate*[Title/Abstract] OR glucose[Title/Abstract] OR fructose[Title/Abstract] OR sucrose[Title/Abstract] OR maltose[Title/Abstract] OR maltodextrin[Title/Abstract] OR dextrin*[Title/Abstract] OR "glucose polymer"[Title/Abstract] OR "glucose polymers"[Title/Abstract] OR "multiple transportable carbohydrate"[Title/Abstract] OR "multiple transportable carbohydrates"[Title/Abstract] OR "carbohydrate supplementation"[Title/Abstract] OR "carbohydrate ingestion"[Title/Abstract] OR "carbohydrate feeding"[Title/Abstract])<br>AND<br>("Electrolytes"[MeSH Terms] OR "Sodium"[MeSH Terms] OR "Potassium"[MeSH Terms] OR "Chlorides"[MeSH Terms] OR "Magnesium"[MeSH Terms] OR "Calcium"[MeSH Terms] OR "Water-Electrolyte Balance"[MeSH Terms] OR "Drinking"[MeSH Terms] OR "Fluid Therapy"[MeSH Terms] OR electrolyte*[Title/Abstract] OR sodium[Title/Abstract] OR potassium[Title/Abstract] OR chloride[Title/Abstract] OR magnesium[Title/Abstract] OR calcium[Title/Abstract] OR salt[Title/Abstract] OR salts[Title/Abstract] OR hydration[Title/Abstract] OR rehydration[Title/Abstract] OR "fluid replacement"[Title/Abstract] OR "fluid intake"[Title/Abstract] OR "fluid ingestion"[Title/Abstract])) OR ("carbohydrate-electrolyte"[Title/Abstract] OR "carbohydrate electrolyte"[Title/Abstract] OR "carbohydrate-electrolyte solution"[Title/Abstract] OR "carbohydrate electrolyte solution"[Title/Abstract] OR "carbohydrate-electrolyte solutions"[Title/Abstract] OR "carbohydrate electrolyte solutions"[Title/Abstract] OR "carbohydrate-electrolyte beverage"[Title/Abstract] OR "carbohydrate electrolyte beverage"[Title/Abstract] OR "carbohydrate-electrolyte beverages"[Title/Abstract] OR "carbohydrate electrolyte beverages"[Title/Abstract] OR "carbohydrate-electrolyte drink"[Title/Abstract] OR "carbohydrate electrolyte drink"[Title/Abstract] OR "carbohydrate-electrolyte drinks"[Title/Abstract] OR "carbohydrate electrolyte drinks"[Title/Abstract] OR "sports drink"[Title/Abstract] OR "sports drinks"[Title/Abstract] OR "sports beverage"[Title/Abstract] OR "sports beverages"[Title/Abstract]))<br>AND<br>("Exercise"[MeSH Terms] OR "Sports"[MeSH Terms] OR "Athletic Performance"[MeSH Terms] OR "Physical Endurance"[MeSH Terms] OR "Running"[MeSH Terms] OR "Bicycling"[MeSH Terms] OR "Swimming"[MeSH Terms] OR "Resistance Training"[MeSH Terms] OR "Muscle Strength"[MeSH Terms] OR "Fatigue"[MeSH Terms] OR exercise[Title/Abstract] OR sport[Title/Abstract] OR sports[Title/Abstract] OR athlete[Title/Abstract] OR athletes[Title/Abstract] OR athletic[Title/Abstract] OR performance[Title/Abstract] OR "exercise performance"[Title/Abstract] OR "athletic performance"[Title/Abstract] OR "sports performance"[Title/Abstract] OR "physical performance"[Title/Abstract] OR "sport-specific performance"[Title/Abstract] OR "exercise capacity"[Title/Abstract] OR "work capacity"[Title/Abstract] OR endurance[Title/Abstract] OR "endurance performance"[Title/Abstract] OR "physical endurance"[Title/Abstract] OR "time to exhaustion"[Title/Abstract] OR "time-to-exhaustion"[Title/Abstract] OR "time trial"[Title/Abstract] OR "time-trial"[Title/Abstract] OR "running performance"[Title/Abstract] OR "cycling performance"[Title/Abstract] OR "swimming performance"[Title/Abstract] OR "power output"[Title/Abstract] OR "mean power"[Title/Abstract] OR "peak power"[Title/Abstract] OR "average power"[Title/Abstract] OR "maximal power"[Title/Abstract] OR "anaerobic power"[Title/Abstract] OR "total work"[Title/Abstract] OR "work completed"[Title/Abstract] OR	**27741**

Database, platform, and search date	Retrospectively adapted database-specific reporting syntax	Records retrieved
	"distance covered"[Title/Abstract] OR "distance completed"[Title/Abstract] OR running[Title/Abstract] OR cycling[Title/Abstract] OR bicycling[Title/Abstract] OR swimming[Title/Abstract] OR sprint[Title/Abstract] OR sprints[Title/Abstract] OR sprinting[Title/Abstract] OR "sprint performance"[Title/Abstract] OR "sprint time"[Title/Abstract] OR "repeated sprint"[Title/Abstract] OR "repeated-sprint"[Title/Abstract] OR "repeated sprint performance"[Title/Abstract] OR "repeated-sprint performance"[Title/Abstract] OR intermittent[Title/Abstract] OR "intermittent exercise"[Title/Abstract] OR "intermittent exercise performance"[Title/Abstract] OR "intermittent exercise capacity"[Title/Abstract] OR "shuttle run"[Title/Abstract] OR "shuttle running"[Title/Abstract] OR "Yo-Yo test"[Title/Abstract] OR "team sport"[Title/Abstract] OR "team sports"[Title/Abstract] OR soccer[Title/Abstract] OR football[Title/Abstract] OR basketball[Title/Abstract] OR tennis[Title/Abstract] OR rugby[Title/Abstract] OR hockey[Title/Abstract] OR "skill performance"[Title/Abstract] OR "skill accuracy"[Title/Abstract] OR accuracy[Title/Abstract] OR "shooting accuracy"[Title/Abstract] OR "passing accuracy"[Title/Abstract] OR "hitting accuracy"[Title/Abstract] OR agility[Title/Abstract] OR "agility performance"[Title/Abstract] OR strength[Title/Abstract] OR "muscle strength"[Title/Abstract] OR "muscular strength"[Title/Abstract] OR "muscle power"[Title/Abstract] OR "muscular power"[Title/Abstract] OR "muscular endurance"[Title/Abstract] OR "resistance exercise"[Title/Abstract] OR "resistance training"[Title/Abstract] OR "jump performance"[Title/Abstract] OR "vertical jump"[Title/Abstract] OR "countermovement jump"[Title/Abstract] OR Wingate[Title/Abstract] OR "Wingate test"[Title/Abstract] OR fatigue[Title/Abstract] OR "muscle fatigue"[Title/Abstract] OR "fatigue index"[Title/Abstract]))<br>NOT<br>("Animals"[MeSH Terms] NOT "Humans"[MeSH Terms])	
**Embase**<br>Platform: Embase.com (Elsevier)<br>Date searched: 27 May 2026	(((('carbohydrate'/exp OR 'glucose'/exp OR 'fructose'/exp OR 'sucrose'/exp OR 'maltose'/exp OR 'maltodextrin'/exp OR (carbohydrate* OR glucose OR fructose OR sucrose OR maltose OR maltodextrin OR dextrin* OR "glucose polymer" OR "glucose polymers" OR "multiple transportable carbohydrate" OR "multiple transportable carbohydrates" OR "carbohydrate supplementation" OR "carbohydrate ingestion" OR "carbohydrate feeding"):ti,ab,kw)<br>AND<br>('electrolyte'/exp OR 'sodium'/exp OR 'potassium'/exp OR 'chloride'/exp OR 'magnesium'/exp OR 'calcium'/exp OR 'hydration'/exp OR 'rehydration'/exp OR (electrolyte* OR sodium OR potassium OR chloride OR magnesium OR calcium OR salt OR salts OR hydration OR rehydration OR "fluid replacement" OR "fluid intake" OR "fluid ingestion"):ti,ab,kw)) OR (("carbohydrate-electrolyte" OR "carbohydrate electrolyte" OR "carbohydrate-electrolyte solution" OR "carbohydrate electrolyte solution" OR "carbohydrate-electrolyte solutions" OR "carbohydrate electrolyte solutions" OR "carbohydrate-electrolyte beverage" OR "carbohydrate electrolyte beverage" OR "carbohydrate-electrolyte beverages" OR "carbohydrate electrolyte beverages" OR "carbohydrate-electrolyte drink" OR "carbohydrate electrolyte drink" OR "carbohydrate-electrolyte drinks" OR "carbohydrate electrolyte drinks" OR "sports drink" OR "sports drinks" OR "sports beverage" OR "sports beverages"):ti,ab,kw))<br>AND<br>('exercise'/exp OR 'sport'/exp OR 'athletic performance'/exp OR 'endurance'/exp OR 'running'/exp OR 'cycling'/exp OR 'swimming'/exp OR 'resistance training'/exp OR 'muscle strength'/exp OR 'fatigue'/exp OR (exercise OR sport OR sports OR athlete OR athletes OR athletic OR performance OR "exercise performance" OR "athletic performance" OR "sports performance" OR	**30240**

Database, platform, and search date	Retrospectively adapted database-specific reporting syntax	Records retrieved
	"physical performance" OR "sport-specific performance" OR "exercise capacity" OR "work capacity" OR endurance OR "endurance performance" OR "physical endurance" OR "time to exhaustion" OR "time-to-exhaustion" OR "time trial" OR "time-trial" OR "running performance" OR "cycling performance" OR "swimming performance" OR "power output" OR "mean power" OR "peak power" OR "average power" OR "maximal power" OR "anaerobic power" OR "total work" OR "work completed" OR "distance covered" OR "distance completed" OR running OR cycling OR bicycling OR swimming OR sprint OR sprints OR sprinting OR "sprint performance" OR "sprint time" OR "repeated sprint" OR "repeated-sprint" OR "repeated sprint performance" OR "repeated-sprint performance" OR intermittent OR "intermittent exercise" OR "intermittent exercise performance" OR "intermittent exercise capacity" OR "shuttle run" OR "shuttle running" OR "Yo-Yo test" OR "team sport" OR "team sports" OR soccer OR football OR basketball OR tennis OR rugby OR hockey OR "skill performance" OR "skill accuracy" OR accuracy OR "shooting accuracy" OR "passing accuracy" OR "hitting accuracy" OR agility OR "agility performance" OR strength OR "muscle strength" OR "muscular strength" OR "muscle power" OR "muscular power" OR "muscular endurance" OR "resistance exercise" OR "resistance training" OR "jump performance" OR "vertical jump" OR "countermovement jump" OR Wingate OR "Wingate test" OR fatigue OR "muscle fatigue" OR "fatigue index"):ti,ab,kw))<br>NOT<br>((('animal'/exp NOT 'human'/exp) OR (animal OR animals OR animal* OR rat OR rats OR mouse OR mice OR murine OR rodent OR rodents OR rabbit OR rabbits OR dog OR dogs OR canine OR pig OR pigs OR swine OR porcine OR cattle OR bovine OR cow OR cows OR sheep OR ovine OR goat OR goats OR horse OR horses OR equine OR fish OR fishes OR zebrafish OR bird OR birds OR avian OR poultry OR chicken OR chickens OR reptile OR reptiles OR amphibian OR amphibians OR insect OR insects OR drosophila):ti,ab,kw))	
**Web of Science Core Collection**<br>Platform: Web of Science (Clarivate)<br>Date searched: 27 May 2026	TS=(((((carbohydrate* OR glucose OR fructose OR sucrose OR maltose OR maltodextrin OR dextrin* OR "glucose polymer" OR "glucose polymers" OR "multiple transportable carbohydrate" OR "multiple transportable carbohydrates" OR "carbohydrate supplementation" OR "carbohydrate ingestion" OR "carbohydrate feeding")<br>AND<br>(electrolyte* OR sodium OR potassium OR chloride OR magnesium OR calcium OR salt OR salts OR hydration OR rehydration OR "fluid replacement" OR "fluid intake" OR "fluid ingestion")) OR ("carbohydrate-electrolyte" OR "carbohydrate electrolyte" OR "carbohydrate-electrolyte solution" OR "carbohydrate electrolyte solution" OR "carbohydrate-electrolyte solutions" OR "carbohydrate electrolyte solutions" OR "carbohydrate-electrolyte beverage" OR "carbohydrate electrolyte beverage" OR "carbohydrate-electrolyte beverages" OR "carbohydrate electrolyte beverages" OR "carbohydrate-electrolyte drink" OR "carbohydrate electrolyte drink" OR "carbohydrate-electrolyte drinks" OR "carbohydrate electrolyte drinks" OR "sports drink" OR "sports drinks" OR "sports beverage" OR "sports beverages"))<br>AND<br>(exercise OR sport OR sports OR athlete OR athletes OR athletic OR performance OR "exercise performance" OR "athletic performance" OR "sports performance" OR "physical performance" OR "sport-specific performance" OR "exercise capacity" OR "work capacity" OR endurance OR "endurance performance" OR "physical endurance" OR "time to exhaustion" OR "time-to-exhaustion" OR "time trial" OR "time-trial" OR "running performance" OR "cycling performance" OR "swimming performance" OR "power output" OR "mean power" OR "peak power" OR "average	**42394**

Database, platform, and search date	Retrospectively adapted database-specific reporting syntax	Records retrieved
	power" OR "maximal power" OR "anaerobic power" OR "total work" OR "work completed" OR "distance covered" OR "distance completed" OR running OR cycling OR bicycling OR swimming OR sprint OR sprints OR sprinting OR "sprint performance" OR "sprint time" OR "repeated sprint" OR "repeated-sprint" OR "repeated sprint performance" OR "repeated-sprint performance" OR intermittent OR "intermittent exercise" OR "intermittent exercise performance" OR "intermittent exercise capacity" OR "shuttle run" OR "shuttle running" OR "Yo-Yo test" OR "team sport" OR "team sports" OR soccer OR football OR basketball OR tennis OR rugby OR hockey OR "skill performance" OR "skill accuracy" OR accuracy OR "shooting accuracy" OR "passing accuracy" OR "hitting accuracy" OR agility OR "agility performance" OR strength OR "muscle strength" OR "muscular strength" OR "muscle power" OR "muscular power" OR "muscular endurance" OR "resistance exercise" OR "resistance training" OR "jump performance" OR "vertical jump" OR "countermovement jump" OR Wingate OR "Wingate test" OR fatigue OR "muscle fatigue" OR "fatigue index")))<br>NOT<br>TS=(animal* OR nonhuman OR "non-human" OR "animal model" OR "animal models" OR "animal experiment" OR "animal experiments" OR "animal study" OR "animal studies" OR veterinary OR livestock OR cattle OR cow OR cows OR bovine OR calf OR calves OR bull OR bulls OR ox OR sheep OR lamb OR lambs OR goat OR goats OR swine OR pig OR pigs OR porcine OR boar OR boars OR horse OR horses OR equine OR donkey OR donkeys OR mule OR mules OR dog OR dogs OR canine OR cat OR cats OR feline OR rabbit OR rabbits OR hare OR hares OR rodent OR rodents OR rat OR rats OR mouse OR mice OR murine OR hamster OR hamsters OR gerbil OR gerbils OR "guinea pig" OR "guinea pigs" OR primate OR primates OR monkey OR monkeys OR macaque OR macaques OR baboon OR baboons OR chimpanzee OR chimpanzees OR fish OR fishes OR zebrafish OR salmon OR trout OR carp OR tilapia OR amphibian OR amphibians OR frog OR frogs OR reptile OR reptiles OR lizard OR lizards OR snake OR snakes OR bird OR birds OR avian OR chicken OR chickens OR poultry OR duck OR ducks OR goose OR geese OR insect OR insects OR drosophila OR fly OR flies OR bee OR bees OR larvae OR larva OR worm OR worms OR nematode OR nematodes OR "Caenorhabditis elegans")	
**Cochrane Central Register of Controlled Trials (CENTRAL)**<br>Platform: Cochrane Library<br>Date searched: 28 May 2026	#1 ([mh Carbohydrates] OR [mh Glucose] OR [mh Fructose] OR [mh Sucrose] OR [mh Maltose] OR [mh Dextrins])<br>#2 (carbohydrate* OR glucose OR fructose OR sucrose OR maltose OR maltodextrin OR dextrin* OR "glucose polymer" OR "glucose polymers" OR "multiple transportable carbohydrate" OR "multiple transportable carbohydrates" OR "carbohydrate supplementation" OR "carbohydrate ingestion" OR "carbohydrate feeding"):ti,ab,kw<br>#3 #1 OR #2<br>#4 ([mh Electrolytes] OR [mh Sodium] OR [mh Potassium] OR [mh Chlorides] OR [mh Magnesium] OR [mh Calcium] OR [mh "Water-Electrolyte Balance"] OR [mh Drinking] OR [mh "Fluid Therapy"])<br>#5 (electrolyte* OR sodium OR potassium OR chloride OR magnesium OR calcium OR salt OR salts OR hydration OR rehydration OR "fluid replacement" OR "fluid intake" OR "fluid ingestion"):ti,ab,kw<br>#6 #4 OR #5<br>#7 ("carbohydrate-electrolyte" OR "carbohydrate electrolyte" OR "carbohydrate-electrolyte solution" OR "carbohydrate electrolyte solution" OR "carbohydrate-electrolyte solutions" OR "carbohydrate electrolyte solutions" OR "carbohydrate-electrolyte beverage" OR "carbohydrate electrolyte beverage" OR "carbohydrate-electrolyte beverages" OR "carbohydrate electrolyte	**4280**

Database, platform, and search date	Retrospectively adapted database-specific reporting syntax	Records retrieved
	beverages" OR "carbohydrate-electrolyte drink" OR "carbohydrate electrolyte drink" OR "carbohydrate-electrolyte drinks" OR "carbohydrate electrolyte drinks" OR "sports drink" OR "sports drinks" OR "sports beverage" OR "sports beverages"):ti,ab,kw<br>#8 (#3 AND #6) OR #7<br>#9 ([mh Exercise] OR [mh Sports] OR [mh "Athletic Performance"] OR [mh "Physical Endurance"] OR [mh Running] OR [mh Bicycling] OR [mh Swimming] OR [mh "Resistance Training"] OR [mh "Muscle Strength"] OR [mh Fatigue])<br>#10 (exercise OR sport OR sports OR athlete OR athletes OR athletic OR performance OR "exercise performance" OR "athletic performance" OR "sports performance" OR "physical performance" OR "sport-specific performance" OR "exercise capacity" OR "work capacity" OR endurance OR "endurance performance" OR "physical endurance" OR "time to exhaustion" OR "time-to-exhaustion" OR "time trial" OR "time-trial" OR "running performance" OR "cycling performance" OR "swimming performance" OR "power output" OR "mean power" OR "peak power" OR "average power" OR "maximal power" OR "anaerobic power" OR "total work" OR "work completed" OR "distance covered" OR "distance completed" OR running OR cycling OR bicycling OR swimming OR sprint OR sprints OR sprinting OR "sprint performance" OR "sprint time" OR "repeated sprint" OR "repeated-sprint" OR "repeated sprint performance" OR "repeated-sprint performance" OR intermittent OR "intermittent exercise" OR "intermittent exercise performance" OR "intermittent exercise capacity" OR "shuttle run" OR "shuttle running" OR "Yo-Yo test" OR "team sport" OR "team sports" OR soccer OR football OR basketball OR tennis OR rugby OR hockey OR "skill performance" OR "skill accuracy" OR accuracy OR "shooting accuracy" OR "passing accuracy" OR "hitting accuracy" OR agility OR "agility performance" OR strength OR "muscle strength" OR "muscular strength" OR "muscle power" OR "muscular power" OR "muscular endurance" OR "resistance exercise" OR "resistance training" OR "jump performance" OR "vertical jump" OR "countermovement jump" OR Wingate OR "Wingate test" OR fatigue OR "muscle fatigue" OR "fatigue index"):ti,ab,kw<br>#11 #9 OR #10<br>#12 #8 AND #11<br>#13 (animal OR animals OR animal* OR rat OR rats OR mouse OR mice OR murine OR rodent OR rodents OR rabbit OR rabbits OR dog OR dogs OR canine OR pig OR pigs OR swine OR porcine OR cattle OR bovine OR cow OR cows OR sheep OR ovine OR goat OR goats OR horse OR horses OR equine OR fish OR fishes OR zebrafish OR bird OR birds OR avian OR poultry OR chicken OR chickens OR reptile OR reptiles OR amphibian OR amphibians OR insect OR insects OR drosophila):ti,ab,kw<br>#14 #12 NOT #13	
**Scopus**<br>Platform: Scopus (Elsevier)<br>Date searched: 28 May 2026	TITLE-ABS-KEY(((((carbohydrate* OR glucose OR fructose OR sucrose OR maltose OR maltodextrin OR dextrin* OR "glucose polymer" OR "glucose polymers" OR "multiple transportable carbohydrate" OR "multiple transportable carbohydrates" OR "carbohydrate supplementation" OR "carbohydrate ingestion" OR "carbohydrate feeding")<br>AND<br>(electrolyte* OR sodium OR potassium OR chloride OR magnesium OR calcium OR salt OR salts OR hydration OR rehydration OR "fluid replacement" OR "fluid intake" OR "fluid ingestion")) OR ("carbohydrate-electrolyte" OR "carbohydrate electrolyte" OR "carbohydrate-electrolyte solution" OR "carbohydrate electrolyte solution" OR "carbohydrate-electrolyte solutions" OR "carbohydrate electrolyte solutions" OR "carbohydrate-electrolyte beverage" OR "carbohydrate electrolyte	**30536**

Database, platform, and search date	Retrospectively adapted database-specific reporting syntax	Records retrieved
	beverage" OR "carbohydrate-electrolyte beverages" OR "carbohydrate electrolyte beverages" OR "carbohydrate-electrolyte drink" OR "carbohydrate electrolyte drink" OR "carbohydrate-electrolyte drinks" OR "carbohydrate electrolyte drinks" OR "sports drink" OR "sports drinks" OR "sports beverage" OR "sports beverages"))<br>AND<br>(exercise OR sport OR sports OR athlete OR athletes OR athletic OR performance OR "exercise performance" OR "athletic performance" OR "sports performance" OR "physical performance" OR "sport-specific performance" OR "exercise capacity" OR "work capacity" OR endurance OR "endurance performance" OR "physical endurance" OR "time to exhaustion" OR "time-to-exhaustion" OR "time trial" OR "time-trial" OR "running performance" OR "cycling performance" OR "swimming performance" OR "power output" OR "mean power" OR "peak power" OR "average power" OR "maximal power" OR "anaerobic power" OR "total work" OR "work completed" OR "distance covered" OR "distance completed" OR running OR cycling OR bicycling OR swimming OR sprint OR sprints OR sprinting OR "sprint performance" OR "sprint time" OR "repeated sprint" OR "repeated-sprint" OR "repeated sprint performance" OR "repeated-sprint performance" OR intermittent OR "intermittent exercise" OR "intermittent exercise performance" OR "intermittent exercise capacity" OR "shuttle run" OR "shuttle running" OR "Yo-Yo test" OR "team sport" OR "team sports" OR soccer OR football OR basketball OR tennis OR rugby OR hockey OR "skill performance" OR "skill accuracy" OR accuracy OR "shooting accuracy" OR "passing accuracy" OR "hitting accuracy" OR agility OR "agility performance" OR strength OR "muscle strength" OR "muscular strength" OR "muscle power" OR "muscular power" OR "muscular endurance" OR "jump performance" OR "vertical jump" OR "countermovement jump" OR Wingate OR "Wingate test" OR fatigue OR "muscle fatigue" OR "fatigue index")))<br>AND NOT<br>TITLE-ABS-KEY(animal OR animals OR animal* OR rat OR rats OR mouse OR mice OR murine OR rodent OR rodents OR rabbit OR rabbits OR dog OR dogs OR canine OR pig OR pigs OR swine OR porcine OR cattle OR bovine OR cow OR cows OR sheep OR ovine OR goat OR goats OR horse OR horses OR equine OR fish OR fishes OR zebrafish OR bird OR birds OR avian OR poultry OR chicken OR chickens OR reptile OR reptiles OR amphibian OR amphibians OR insect OR insects OR drosophila)	

Reporting note: The database-specific field tags and controlled-vocabulary syntax shown above were retrospectively adapted from the original broad concept blocks to improve reporting transparency; they are not a verbatim exported search history. The searches were not rerun with these reconstructed strings. The record counts are those returned by the original searches conducted on 27–28 May 2026.

Supplementary Material S2 (PRISMA 2020 Checklist)

Section and Topic	Item	Checklist item	Location where item is reported
**TITLE**			
Title	1	Identify the report as a systematic review.	√ Title page
**ABSTRACT**			
Abstract	2	See the PRISMA 2020 for Abstracts checklist.	√ Page 2 Abstract
**INTRODUCTION**			
Rationale	3	Describe the rationale for the review in the context of existing knowledge.	√ Pages 3 to 5 Section 1
Objectives	4	Provide an explicit statement of the objective(s) or question(s) the review addresses.	√ Page 5 Section 1
**METHODS**			
Eligibility criteria	5	Specify the inclusion and exclusion criteria for the review and how studies were grouped for the syntheses.	√ Pages 6 to 7 Section 2.2
Information sources	6	Specify all databases, registers, websites, organisations, reference lists and other sources searched or consulted to identify studies. Specify the date when each source was last searched or consulted.	√ Page 6 Section 2.1
Search strategy	7	Present the full search strategies for all databases, registers and websites, including any filters and limits used.	√ Supplementary Appendix S1
Selection process	8	Screening procedures, including the use of ASReview and independent full-text review with conflict resolution, are described.	√ Pages 7 to 8 Section 2.3
Data collection process	9	Specify the methods used to collect data from reports, including how many reviewers collected data from each report, whether they worked independently, any processes for obtaining or confirming data from study investigators, and if applicable, details of automation tools used in the process.	√ Page 8 Section 2.4
Data items	10a	All eligible exercise-performance outcomes, including endurance, sprint, power, work, distance, and strength outcomes, are listed and defined.	√ Page 8 Section 2.4
	10b	Additional variables, including study design, participant characteristics, CHO-E supplementation, exercise protocols, ambient temperature, and assumptions for unavailable correlations, are described.	√ Pages 8 to 10 Sections 2.4 and 2.6.1
Study risk of bias assessment	11	Specify the methods used to assess risk of bias in the included studies, including details of the tool(s) used, how many reviewers assessed each study and whether they worked independently, and if applicable, details of automation tools used in the process.	√ Pages 8 to 9 Section 2.5
Effect measures	12	Specify for each outcome the effect measure(s) (e.g. risk ratio, mean difference) used in the synthesis or presentation of results.	√ Pages 9 to 12 Section 2.6.1
Synthesis methods	13a	Procedures for determining study eligibility for each synthesis are described.	√ Pages 6 to 14 Sections 2.2 and 2.6

Section and Topic	Item	Checklist item	Location where item is reported
	13b	Methods for handling missing data, calculating effect sizes, accounting for dependent effects, and standardizing effect directions are described.	√ Pages 8 to 12 Sections 2.4 and 2.6.1
	13c	Study selection, risk of bias, individual effects, pooled effects, subgroup results, meta-regression, publication bias, and sensitivity analyses are presented using tables and figures.	√ Pages 12 to 15 Sections 2.6 and 2.7
	13d	Three-level random-effects meta-analyses using metafor are described, including REML estimation, variance-covariance modelling, heterogeneity statistics, prediction intervals, and R software.	√ Pages 12 to 14 Sections 2.6.2 and 2.6.3
	13e	Describe any methods used to explore possible causes of heterogeneity among study results (e.g. subgroup analysis, meta-regression).	√ Pages 13 to 14 Section 2.6.3
	13f	Describe any sensitivity analyses conducted to assess robustness of the synthesized results.	√ Pages 13 to 15 Sections 2.6.2 and 2.7
Reporting bias assessment	14	Describe any methods used to assess risk of bias due to missing results in a synthesis (arising from reporting biases).	√ Pages 14 to 15 Section 2.7
Certainty assessment	15	Describe any methods used to assess certainty (or confidence) in the body of evidence for an outcome.	√ Page 15 Section 2.8; Supplementary S4
**RESULTS**			
Study selection	16a	Describe the results of the search and selection process, from the number of records identified in the search to the number of studies included in the review, ideally using a flow diagram.	√ Pages 16 to 18 Section 3.1; Figure 1
	16b	Cite studies that might appear to meet the inclusion criteria, but which were excluded, and explain why they were excluded.	√ Page 17 Section 3.1; Figure 1
Study characteristics	17	Cite each included study and present its characteristics.	√ Pages 17 to 19 Sections 3.1 and 3.2; Supplementary S3
Risk of bias in studies	18	Present assessments of risk of bias for each included study.	√ Pages 19 to 20 Section 3.3; Figure 2
Results of individual studies	19	For all outcomes, present, for each study: (a) summary statistics for each group (where appropriate) and (b) an effect estimate and its precision (e.g. confidence/credible interval), ideally using structured tables or plots.	√ Pages 20 to 23 Sections 3.4 and 3.5; Figures 3 and 4
Results of syntheses	20a	For each synthesis, briefly summarise the characteristics and risk of bias among contributing studies.	√ Pages 18 to 23 Sections 3.2 to 3.5
	20b	Present results of all statistical syntheses conducted. If meta-analysis was done, present for each the summary estimate and its precision (e.g. confidence/credible interval) and measures of statistical heterogeneity. If comparing groups, describe the direction of the effect.	√ Pages 20 to 24 Sections 3.4 and 3.5; Figures 3 to 5

Section and Topic	Item	Checklist item	Location where item is reported
	20c	Present results of all investigations of possible causes of heterogeneity among study results.	√ Pages 22 to 24 Section 3.5; Figures 4 and 5
	20d	Present results of all sensitivity analyses conducted to assess the robustness of the synthesized results.	√ Pages 24 to 25 Section 3.6; Supplementary S6 and S7
Reporting biases	21	Present assessments of risk of bias due to missing results (arising from reporting biases) for each synthesis assessed.	√ Pages 19 to 20 Section 3.3; Supplementary S5
Certainty of evidence	22	Present assessments of certainty (or confidence) in the body of evidence for each outcome assessed.	√ Pages 21 to 23 Sections 3.4 and 3.5; Supplementary S4
**DISCUSSION**			
Discussion	23a	Provide a general interpretation of the results in the context of other evidence.	√ Pages 25 to 27 Sections 4.1 to 4.4
	23b	Discuss any limitations of the evidence included in the review.	√ Pages 27 to 28 Section 4.6
	23c	Discuss any limitations of the review processes used.	√ Pages 27 to 28 Section 4.6
	23d	Discuss implications of the results for practice, policy, and future research.	√ Pages 28 to 29 Section 4.7 and Section 5
**OTHER INFORMATION**			
Registration and protocol	24a	Provide registration information for the review, including register name and registration number, or state that the review was not registered.	√ Pages 2 and 5 Abstract and Section 2
	24b	Indicate where the review protocol can be accessed, or state that a protocol was not prepared.	√ Page 5 Section 2
	24c	Describe and explain any amendments to information provided at registration or in the protocol.	√ Page 5 Section 2
Support	25	Describe sources of financial or non-financial support for the review, and the role of the funders or sponsors in the review.	√ Page 30 Funding
Competing interests	26	Declare any competing interests of review authors.	√ Page 31 Conflicts of Interest

Section and Topic	Item	Checklist item	Location where item is reported
Availability of data, code and other materials	27	Report which of the following are publicly available and where they can be found: template data collection forms; data extracted from included studies; data used for all analyses; analytic code; any other materials used in the review.	√<br>Page 30<br>Data Availability Statement

From: Page MJ, McKenzie JE, Bossuyt PM, Boutron I, Hoffmann TC, Mulrow CD, et al. The PRISMA 2020 statement: an updated guideline for reporting systematic reviews. BMJ 2021;372:n71. doi: 10.1136/bmj.n71

Supplementary Material S3 (Study characteristics)

Study	Design (RCT)	Participants		Age (years)	Test	supplementation protocol		Exercise Protocol	Ambient temperature (℃)	outcomes	Summary of Reported Findings
		Sample (M/F)	characteristic			Exercise	Control				
O'Neal et al. (2013)	Crossover	36(23/13)	recreationally-active exercisers	23±3	Wingate	CE: 6%; 3 aliquots at 0/20/40 min during EX; total 847 ± 368 mL;	flavored non-caloric-electrolyte beverage	50-min cycling 60–65% HRR with 5×2-min seated rests; 3×30-s WAnT ~7% BM resistance, 2.5-min passive recovery;	25.1±0.3	Mean power	No significant performance effect
Kalman et al. (2012)	Crossover	12(12/0)	exercise-trained	26.6±5.7	Running	SD: 5–6%CE; ~125% BM loss within 60 min post-DHE, pre-TTE rehydration; 2184 ± 358 mL;	bottled water	DHE: 2×30-min treadmill walk/jog at 2–7 mph, 0% grade, 10-min rest; post-rehydration TTE: treadmill 4.2 mph, grade +2.5% q3min to exhaustion;	22	TTE	No significant performance effect
Lee et al. (2011)	Crossover	12(12/0)	physically active	24.3±1.6	Cycling	CE: 6.8%; q15min during 75-min heat cycling and first 60-min recovery to total ≈150% sweat loss, 2325 ± 434 mL; 1.5 mL/kg in 75-min cycling;	Placebo	75-min and final cycling TTE both at 65% VO_2peak, separated by 5-h recovery;	32.1 ± 0.3	TTE	Extending the time to exhaustion in endurance cycling
Del Coso et al. (2008)	Crossover	7(7/0)	endurance-trained cyclists	NR; adult endurance-trained cyclists	Cycling	CE: 6%; CE: 8%; 2.44 ± 0.13 L replacing ~100% sweat loss; one-third immediately pre-exercise and remaining aliquots at 8/30/60/90 min during 120-min heat cycling;	Mineral water	120-min cycling at 63% VO_2max; right knee-extensor MVC measured immediately before and after cycling (3×4s)；	36±1	MVC	Effectively maintain maximum voluntary muscle contraction force
Liang et al. (2024)	Crossover	14(14/0)	trained	20 ± 2	Running	CE: 6%; 150 mL at 5, 10, and 15 km during 21-km treadmill running; total 450 mL;	pure water	21-km treadmill run: 16 km at 65% VO_2max + self-paced final 5-km TT; pre/post CMJ and quadriceps MVIC;	23±1	Time trial、CMJ、MVC	only preserved MVIC, but not 5-km time or CMJ
Criswell et al. (1991)	Cluster-assigned parallel	44(44/0)	high school football players	16±1.0	sprints	GP: 7%; 170 mL at six drink breaks before and during a 50-play football scrimmage; total 1020 mL;	non-nutritive electrolyte artificial sweetener placebo	repeated-sprint test performed before and after scrimmage: 8 maximal-effort 40-yd sprints with 40-sec jog recovery;	28	cumulative sprint Peak speed	No significant performance effect
Ferrauti et al. (1997)	Crossover	16(8/8)	tennis player	22.9 ± 3.4	sprints	CE: 7.6%; 150/100 mL for men/women pre-play and every 15 min at changeovers, plus 400 mL double-concentrated drink during 30-min rest; total 2.8 L/243 g (man) and 2.0 L/182 g CHO (women)	Non-caloric placebo	240-min singles tennis match; 30-min rest after 150 min; post-match TST: 6×15-m sprints, 30-s breaks.	28 ± 2	sprints time	CHO improved TST but not BMT or match success
Millard-Stafford et al. (2005)	Crossover	10(10/0)	highly-trained distance runners	23.7 ± 3.6	Running	CE: 6%; CE: 8%; 400 mL 15 min pre-run, then 250 mL every 5 km during 32-km run; total planned intake ≈1.9 L;	Artificially sweetened placebo	32-km outdoor run; marathon pace except race efforts at 14.4–16.0 km and final 5 km;	25.6	Time trial	Significantly shorten the completion time for the last 5 km
Johannsen et al. (2013)	Crossover	17(17/0)	healthy trained	25.4 ± 4	Cycling	CES: 6 mL/kg; then drink as desired during 2-h cycling; total intake 1952 ± 674 g;	non-caloric-electrolyte placebo	2-h steady-state cycling at 68.4 ± 9.3% VO_2peak, followed immediately by a 30-min cycling performance test;	24.4 ± 1.7	Total work	Significantly improved total work done in 30-minute time trials
Davis et al. (1988)	Crossover	19(19/0)	cyclists	21	Cycling	CE: 2.5%; CE: 6%; 275 mL every 20 min during cycling session, first at 15 min into Task I;	Flavored water placebo	Fixed-work cycling at 75% VO_2max: 2-h-equivalent Task I, 30-min rest, then 30-min-equivalent Task II;	27.15	Time trial	Significantly reduce time in later fixed-load cycling time trials
Gant et al. (2007)	Crossover	9(9/0)	soccer players	22	LIST	CES: 6.2%; 6.5 mL/kg pre-exercise, then 3.5 mL/kg after each 15-min LIST block during rest periods; total intake 1.41 ± 0.21 L, providing 87.9 g CHO;	Flavored water placebo	60-min LIST: 4 × ~15-min blocks, each with 11 × 15-m sprints; ~5-min rest between blocks;	30.4 ± 0.1	cumulative sprint time	Significantly shorten cumulative sprint time
Bergen et al. (1998)	Crossover	16(8/8)	moderately trained subjects	26.6 ± 3.9	Cycling	CE: 8%; 2.5 mL/kg every 15 min during intermittent cycling;	Low-calorie placebo	HIIT: 1-min work/1-min rest at 80% VO_2max, two 45-min sessions separated by 10-min rest; trial stopped at 100 min or earlier by workload/HR/core-temperature criteria;	35 ± 1	Time to exercise termination	Significantly prolong exercise time
Millard-Stafford et al. (1997)	Crossover	12(12/0)	highly trained runners	29.8 ± 6.2	Running	CE: 6%; CE: 8%; 1 L 60 min pre-run, then drink as desired at 7.5 and 13.4 km breaks during 15-km run;	plain water	15-km treadmill run: self-paced first 13.4 km with breaks at 7.5 and 13.4 km, then all-out final 1.6 km;	27.75	Time trial	Significantly reduced the time to complete the final 1.6 km of all-out running.
Gusbakti et al. (2006)	Crossover	10(10/0)	physically active	24.6 ± 0.3	Cycling	MCE: 6%; HCE: 12%; 3 mL/kg; pre-5 min + q20 min during EX;	flavored water placebo	Cycle 60–64% VO2max, 60 rpm; constant-load to fatigue;	31	TTE	significantly extend the time to exhaustion
Atan et al. (2024)	Crossover	16(16/0)	soccer players	15.6 ± 0.4 (study mean)	Running	CE: 6%; 5 mL/kg BM immediately before the 80-min SSP and 2 mL/kg BM during rest periods/every 20 min;	flavored water placebo	80-min test: four 20-min sprint blocks; peak sprint speed by GPS and repeated CMJ;	32.2 ± 2.1	Peak sprint time、CMJ	No significant performance effect

Morris et al. (2003)	Crossover	9(9/0)	university student players	23.3 ± 1.4	Running	CE: 6.5%; 6.5 mL/kg before exercise, then 4.5 mL/kg during each 15-min exercise set plus 4-min rest;	taste placebo	five 15-min intermittent shuttle-running sets (LIST), followed by 60-s rest to exhaustion or rectal temperature >39.5°C;	30.5 ± 0.1	Total distance completed、sprint time	No significant performance effect
Murray et al. (1989)	Crossover	12(7/5)	moderately active adults	30.7 ± 1.4	Cycling	CE: 6%、8%、10%; 2.5 mL/kg before exercise and during each 5-min rest;	sweetened water	3 × 20-min cycling at 65% VO_2max, with 5-min rests; final timed 1200-pedal-revolution cycling task.	33.4 ± 0.2	cycling time-trial	6% CHO-E significantly improved; 8% and 10% were not significant
Millard-Stafford et al. (1992)	Crossover	8(8/0)	distance runners	32.1 ± 6.0	Running	CE: 7%; 400 mL 30 min pre-exercise plus ~250 mL every 5 km during the 40-km run; actual during-run intake ~1.98 L.	sweetened water placebo	Outdoor 40-km run in hot-humid conditions; first 35 km at self-selected training pace and final 5 km at maximal effort;	26.5	Time trial	Significant improvement time
Byrne et al. (2005)	Crossover	14(14/0)	soldiers	20.7 ± 0.8	Running	CE: 5.8%; 5 mL/kg immediately before exercise, then 3 mL/kg every 15 min during exercise and rest; total intake ~2.31 L.	plain water	3 × 60-min loaded treadmill marching at 4.4 km/h and 5% grade with ~14 kg load; separated by 15-min rests;	35	Total marching duration	No significant performance effect
Millard-Stafford et al. (1990)	Crossover	10(10/0)	triathletes	29.6 ± 3.7	Triathlon	CE:7%; 2 mL/kg per feeding, consumed after the swim, every 8 km during cycling, and every 3.2 km during running; total intake was 1480 ± 110 mL;	flavored water placebo	1.5-km swim, 40-km cycling, and 10-km treadmill run at self-selected race pace;	30.0 ± 0.6	Time trial	No significant performance effect
Bilzon et al. (2000)	Crossover	13(13/0)	volunteers	32.3 ± 1.4	Running	CES or placebo consumed during 4-h recovery before T2, with total fluid equal to 140% BM; CES: 6.9%; providing 138 ± 12 g CHO.	flavored water placebo	T1: 60% VO_2max running for up to 90 min or fatigue; after 4 h recovery, T2: same-intensity run to exhaustion;	35.1 ± 0.1	TTE	Significant improvement time to exhaustion

Eligibility note: The age criterion was applied to the study-reported mean age. Del Coso et al. did not report a numerical mean age; the sample was described as endurance-trained cyclists and indexed as adults. Atan et al. reported a mean age of 15.6 ± 0.4 years. Both studies were retained. Atan et al. was a complete, formally published conference-proceedings paper with sufficient randomized methods and extractable numerical data, not an abstract-only record.

Abbreviations: BM, body mass; CE, carbohydrate–electrolyte beverage; CES, carbohydrate–electrolyte solution; CHO, carbohydrate; CMJ, countermovement jump; DHE, dehydration exercise; EX, exercise; GP, 7% glucose-polymer beverage containing electrolytes; HCE, 12% carbohydrate–electrolyte mixture; HRR, heart-rate reserve; LIST, Loughborough Intermittent Shuttle Test; MCE, 6% carbohydrate–electrolyte mixture; MVC/MVIC, maximal voluntary (isometric) contraction; SSP, soccer simulation protocol; TTE, time to exhaustion; WAnT, Wingate anaerobic test.

Supplementary Material S4 (GRADE Assessment)

Outcome	Evidence base	Certainty of Evidence Assessment					Hedges' g [95% CI] *	GRADE†
		Risk of Bias	Inconsistency	Indirectness	Imprecision	Publication bias		
**Secondary overall (omnibus) synthesis**								
Overall exercise performance	21 studies<br>K = 32<br>N = 316	Serious[1]	Not serious<br>(I^2 = 46.7%)	Not serious	Serious[2]<br>(N < 400; CI excludes 0)	No additional downgrade[3]	0.37 [0.21, 0.53] *	⊕⊕◯◯ Low
Aerobic endurance								
Aerobic endurance	15 studies<br>K = 21<br>N = 188	Serious[1]	Not serious<br>(I^2 = 42.9%)	Not serious	Serious[2]<br>(N < 400; CI excludes 0)	No additional downgrade[3]	0.45 [0.26, 0.64] *	⊕⊕◯◯ Low
Anaerobic endurance								
Anaerobic endurance	3 studies<br>K = 3<br>N = 89	Serious[1]	Not serious<br>($I^2 \approx$ 0%)	Not serious	Very serious[2]<br>(N < 400; CI crosses 0)	No additional downgrade[3];<br>formal test unreliable (studies < 10)	0.19 [−0.29, 0.66]	⊕◯◯◯ Very low
Explosive power								
Explosive power	4 studies<br>K = 5<br>N = 55	Serious[1]	Serious<br>(I^2 = 77.1%)	Not serious	Very serious[2]<br>(N < 400; CI crosses 0)	No additional downgrade[3];<br>formal test unreliable (studies < 10)	0.18 [−0.20, 0.55]	⊕◯◯◯ Very low
Strength								
Strength	2 studies<br>K = 3<br>N = 21	Serious[1]	Not serious<br>($I^2 \approx$ 0%)	Not serious	Very serious[2]<br>(N < 400; CI crosses 0)	No additional downgrade[3];<br>formal test unreliable (studies < 10)	0.38 [−0.15, 0.92]	⊕◯◯◯ Very low

Notes: * $p < 0.05$. K, number of effect sizes; N, number of unique participants; CI, confidence interval. Participants in crossover trials and participants contributing multiple effects within one outcome were counted once. [1] Risk of bias was downgraded one level for every outcome because all contributing studies had at least some concerns after reassessment: overall, 19 some concerns/2 high; aerobic endurance, 14/1; anaerobic endurance, 2/1; explosive power, 4/0; strength, 2/0. A two-level risk-of-bias downgrade was not applied because high-risk evidence did not predominate. [2] Imprecision: overall and aerobic endurance were downgraded one level because N was below 400 although the CI excluded 0; the three sparse non-aerobic outcomes were downgraded two levels because N was below 400 and the CI crossed 0 and spanned materially different effects. The N < 400 threshold was used as a heuristic for SMD evidence together with CI width, not as an isolated absolute rule. Inconsistency was downgraded one level only for explosive power (I^2 = 77.1%). Indirectness was not downgraded because populations, interventions, comparators, and direct performance outcomes matched the review question. [3] No additional publication-bias downgrade was made. Cluster-aware asymmetry evidence for the larger syntheses was inconclusive, and formal assessment was unreliable for outcomes with fewer than 10 studies; this was not interpreted as evidence that publication bias was absent. GRADE certainty: High, very confident; Moderate, moderately confident; Low, limited confidence; Very low, very limited confidence.

Supplementary Material S5 (Funnel plot)

Publication-bias and small-study-effects assessment after correction of crossover effect sizes

Dependence is retained, and trim-and-fill is not used as evidence that publication bias is absent.

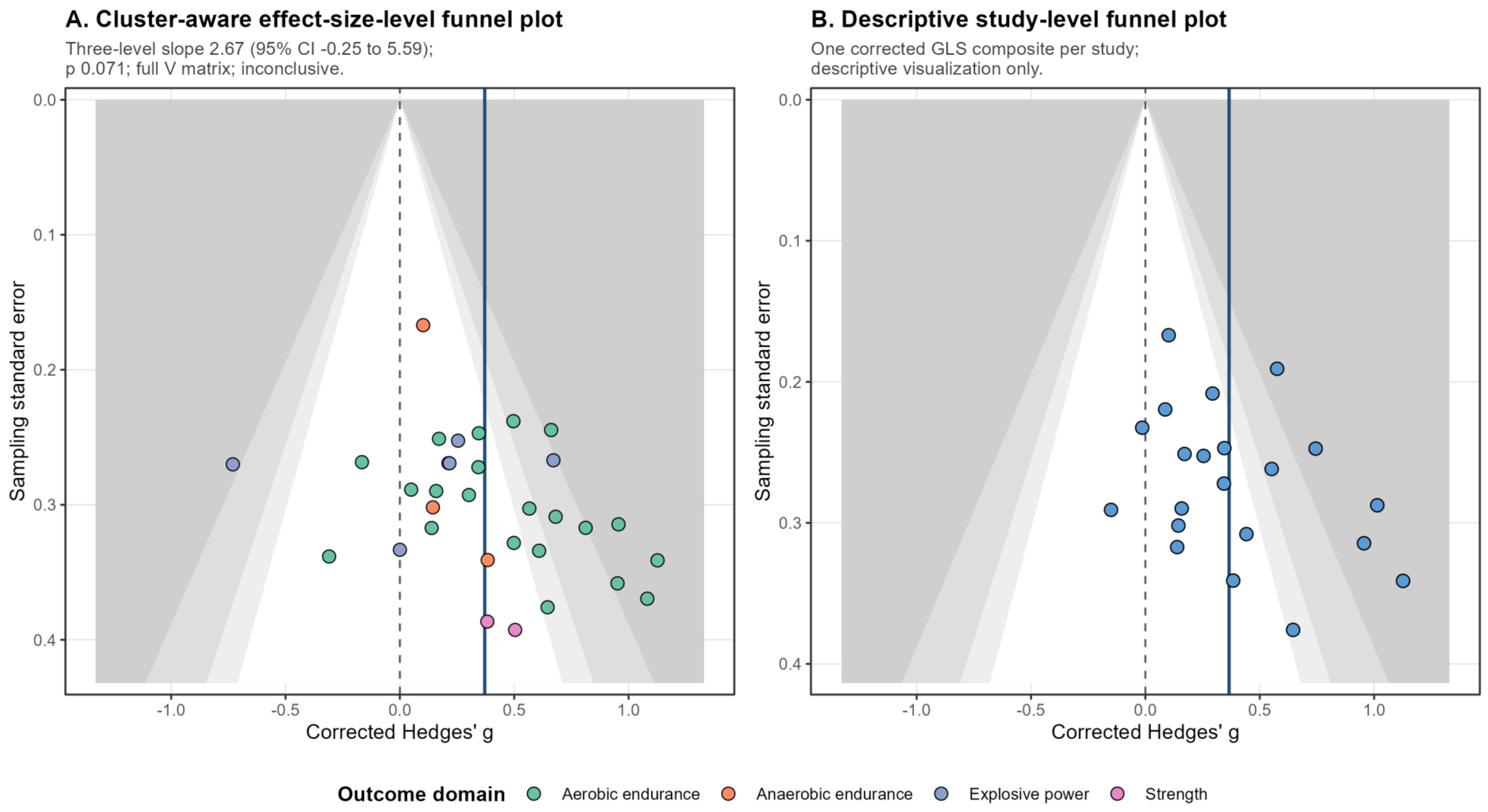

Significance contours around the null: white, p >= 0.10; light grey, 0.05-0.10; medium grey, 0.01-0.05; outer grey, p < 0.01. Solid blue lines show the corresponding corrected pooled estimates.

Supplementary Material S6 (Summary of Sensitivity Analysis Results)

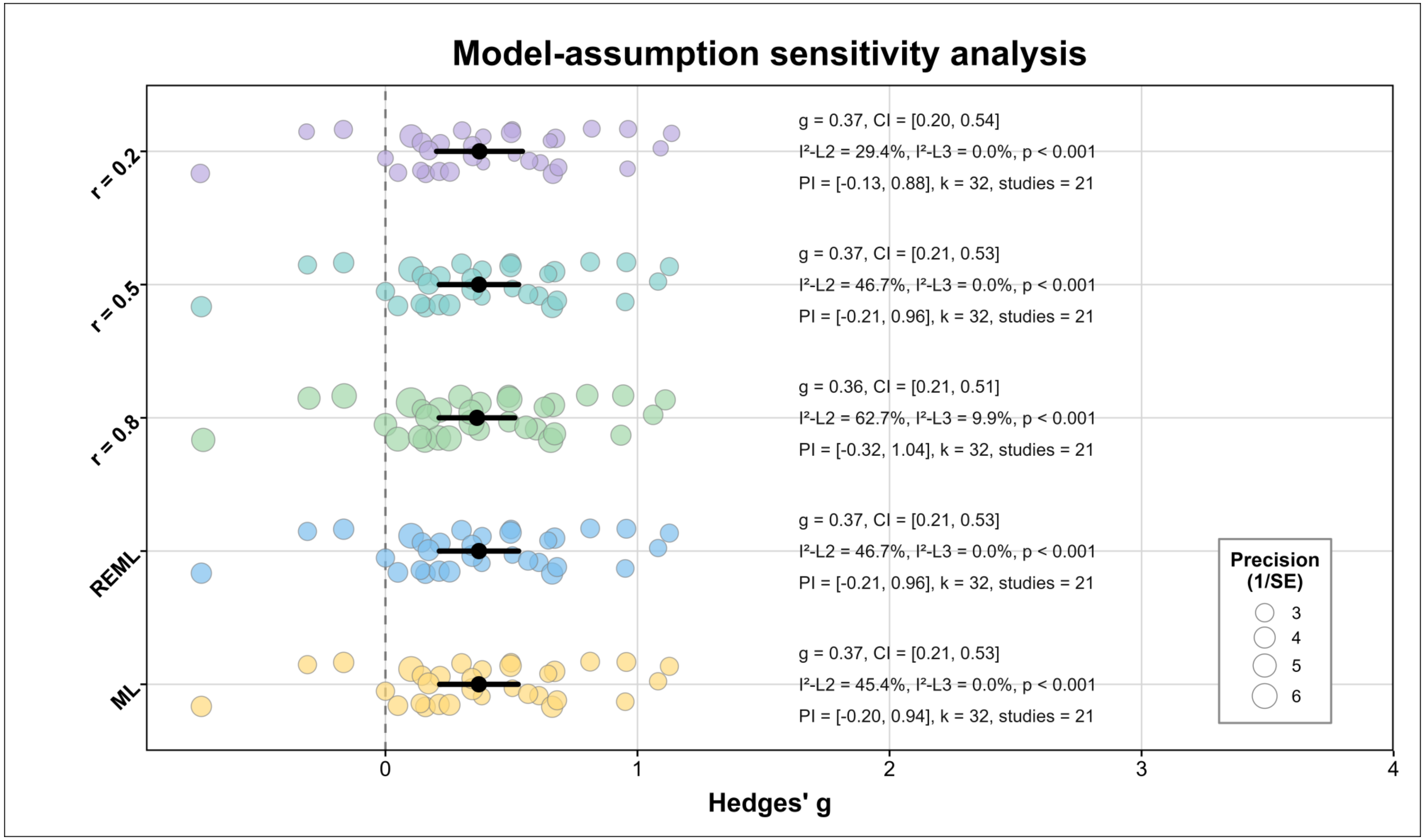

Supplementary Material S7 (A Sensitivity Analysis Based on Level-3 Leave one-out)

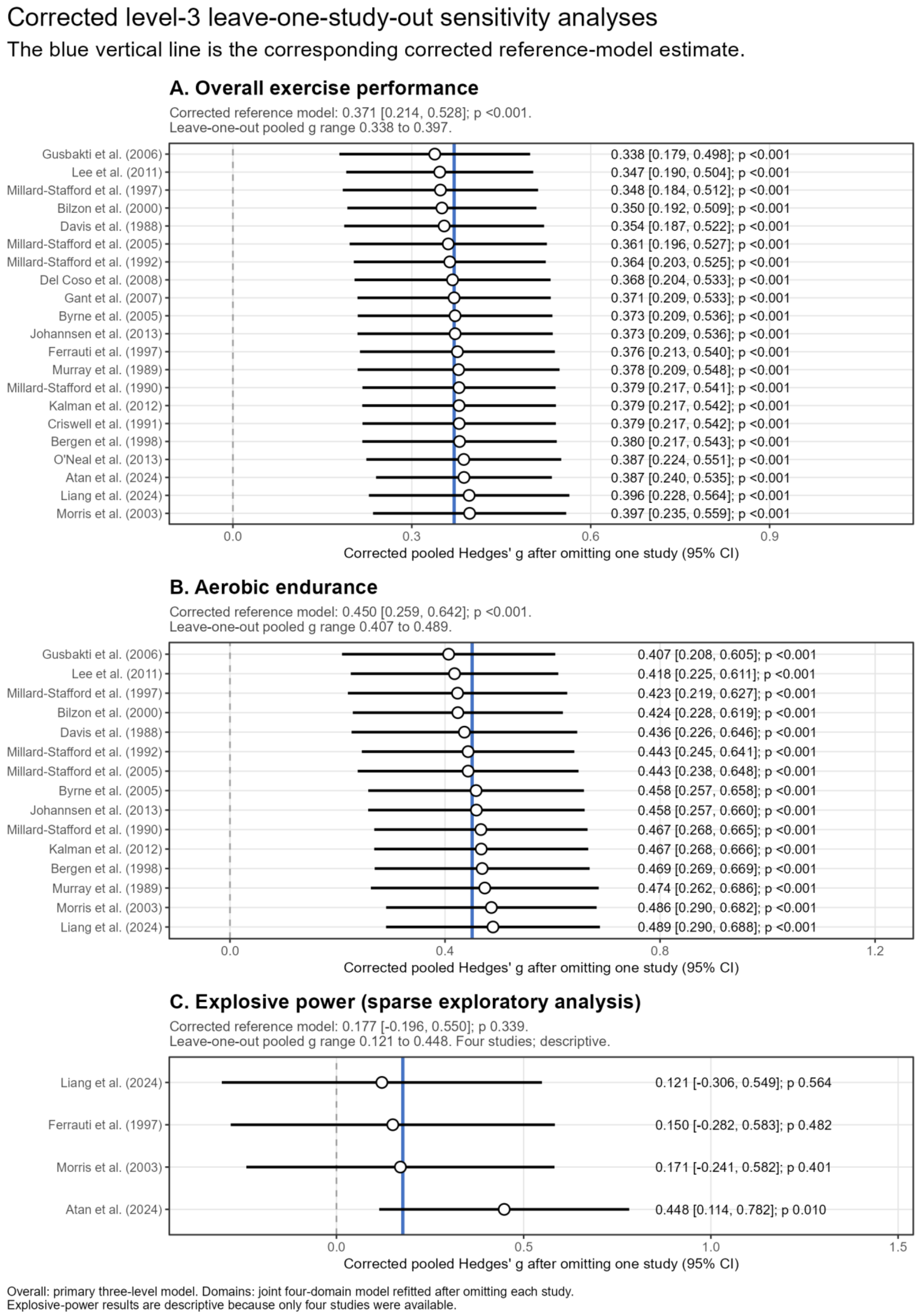

Supplementary Material S8 (Power Visualization)

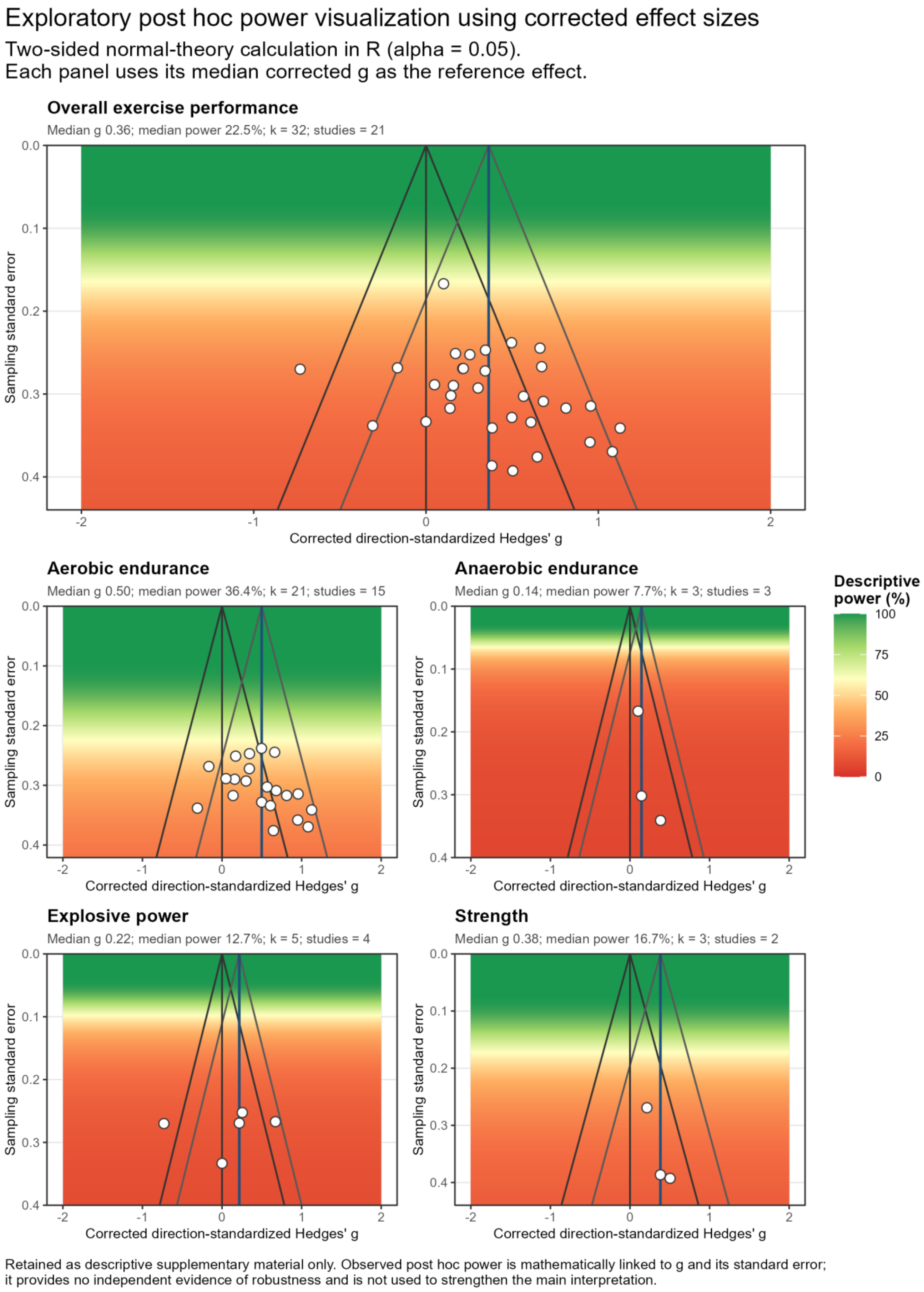

Supplementary Material S9 (Study-level RoB 2 Signalling-Question Responses and Rationales)

Study / design	D1 Randomization	DS Period / carryover	D2 Deviations / analysis	D3 Missing outcome	D4 Outcome measurement	D5 Selection of result	Overall
**O'Neal et al. (2013) (Crossover)**	1.1=NI; 1.2=NI; 1.3=NI → Some concerns. Counterbalanced order reported, but random-sequence generation and concealment were not described.	S1=PY; S2=NA; S3=PY → Low. Counterbalanced trials were separated by 7–14 days (report pp. 2, 5).	2.1=NI; 2.2=NI; 2.3=PN; 2.4=NA; 2.5=NA; 2.6=PY; 2.7=NA → Low. No trial-context deviations were reported; analysis followed assigned condition.	3.1=PY; 3.2–3.4=NA → Low. Nearly all eligible performance data were available; one mechanical failure was a small, non-outcome-dependent loss.	4.1=PN; 4.2=PN; 4.3=NI; 4.4=PN; 4.5=NA → Low. Direct time, distance, power, jump, or force outcomes were recorded with the same protocol/device; assessor judgment was unlikely to alter the recorded value.	5.1=NI; 5.2=PN; 5.3=NI; 5.4=N → Some concerns. No analysis plan finalized before unblinding was verifiable; no clear selective omission was found, but analysis-choice selection could not be excluded.	Some concerns. Least-favourable domain rule.
**Kalman et al. (2012) (Crossover)**	1.1=PY; 1.2=NI; 1.3=NI → Some concerns. Random order was reported; concealment and sequence-generation method were not reported.	S1=NI; S2=NI; S3=PY → Some concerns. Trials were at least 5 days apart, but balance of treatment sequences was not reported (p. 1).	2.1=PN; 2.2=Y; 2.3=PN; 2.4=NA; 2.5=NA; 2.6=PY; 2.7=NA → Low. Participants were blinded but investigators were not; no trial-context deviations were reported.	3.1=PY; 3.2–3.4=NA → Low. Eligible sport-drink versus water performance data were available for nearly all participants.	4.1=PN; 4.2=PN; 4.3=Y; 4.4=PN; 4.5=NA → Low. Direct time, distance, power, jump, or force outcomes were recorded with the same protocol/device; assessor judgment was unlikely to alter the recorded value.	5.1=NI; 5.2=PN; 5.3=NI; 5.4=N → Some concerns. No analysis plan finalized before unblinding was verifiable; no clear selective omission was found, but analysis-choice selection could not be excluded.	Some concerns. Least-favourable domain rule.
**Lee et al. (2011) (Crossover)**	1.1=PY; 1.2=NI; 1.3=NI → Some concerns. Randomized trials were reported; sequence generation and allocation concealment were not described.	S1=NI; S2=NI; S3=PY → Some concerns. Trials were at least 7 days apart, but sequence balance was not reported (p. 4).	2.1=PN; 2.2=PN; 2.3=PN; 2.4=NA; 2.5=NA; 2.6=PY; 2.7=NA → Low. Sports drink and placebo were double-blind; no trial-context deviations were reported.	3.1=PY; 3.2–3.4=NA → Low. Eligible performance results were available for nearly all randomized periods.	4.1=PN; 4.2=PN; 4.3=PN; 4.4=PN; 4.5=NA → Low. Direct time, distance, power, jump, or force outcomes were recorded with the same protocol/device; assessor judgment was unlikely to alter the recorded value.	5.1=NI; 5.2=PN; 5.3=NI; 5.4=N → Some concerns. No analysis plan finalized before unblinding was verifiable; no clear selective omission was found, but analysis-choice selection could not be excluded.	Some concerns. Least-favourable domain rule.
**Del Coso et al. (2008) (Crossover)**	1.1=PY; 1.2=NI; 1.3=NI → Some concerns. Randomized trial order was reported; sequence generation and concealment were not described.	S1=NI; S2=NI; S3=PY → Some concerns. Trials were 48 h–4 d apart; carryover was unlikely for the acute drinks, but sequence balance was not reported (p. 3).	2.1=PY; 2.2=PY; 2.3=PN; 2.4=NA; 2.5=NA; 2.6=PY; 2.7=NA → Low. Beverages were distinguishable, but no trial-context deviations were reported and paired analyses were used.	3.1=PY; 3.2–3.4=NA → Low. Eligible force outcomes were available for all or nearly all randomized periods.	4.1=PN; 4.2=PN; 4.3=NI; 4.4=PN; 4.5=NA → Low. Direct time, distance, power, jump, or force outcomes were recorded with the same protocol/device; assessor judgment was unlikely to alter the recorded value.	5.1=NI; 5.2=PN; 5.3=NI; 5.4=N → Some concerns. No analysis plan finalized before unblinding was verifiable; no clear selective omission was found, but analysis-choice selection could not be excluded.	Some concerns. Least-favourable domain rule.

Study / design	D1 Randomization	DS Period / carryover	D2 Deviations / analysis	D3 Missing outcome	D4 Outcome measurement	D5 Selection of result	Overall
**Liang et al. (2024) (Crossover)**	1.1=PY; 1.2=NI; 1.3=NI → Some concerns. Randomized crossover order was reported; allocation concealment before sequence assignment was not described.	S1=NI; S2=NI; S3=PY → Some concerns. A 1-week washout was used, but sequence balance was not reported (pp. 2–3).	2.1=PN; 2.2=PN; 2.3=PN; 2.4=NA; 2.5=NA; 2.6=PY; 2.7=NA → Low. Opaque coded containers supported blinding; no trial-context deviations were reported.	3.1=PY; 3.2–3.4=NA → Low. Fourteen of 15 participants completed all trials; the single loss was an ankle injury unrelated to beverage assignment.	4.1=PN; 4.2=PN; 4.3=PN; 4.4=PN; 4.5=NA → Low. Direct time, distance, power, jump, or force outcomes were recorded with the same protocol/device; assessor judgment was unlikely to alter the recorded value.	5.1=NI; 5.2=PN; 5.3=NI; 5.4=N → Some concerns. No analysis plan finalized before unblinding was verifiable; no clear selective omission was found, but analysis-choice selection could not be excluded.	Some concerns. Least-favourable domain rule.
**Criswell et al. (1991) (Cluster-assigned parallel)**	1.1=PY; 1.2=NI; 1.3=NI → Some concerns. Treatments were randomized to teams, but concealment and the sequence method were not described. Under the cluster-randomized addendum, participants were identified and teams formed before beverage allocation, so recruitment timing was judged low risk.	S1–S3=NA → Not applicable. Treated as a parallel comparison; assignment occurred at team rather than individual level.	2.1=PN; 2.2=PN; 2.3=PN; 2.4=NA; 2.5=NA; 2.6=N; 2.7=Y → High. Four teams were assigned treatment, but participant-level t tests/ANOVA ignored clustering (2.6=N; 2.7=Y), with likely material impact.	3.1=PY; 3.2–3.4=NA → Low. Eligible sprint outcomes were reported for the randomized treatment groups.	4.1=PN; 4.2=PN; 4.3=PN; 4.4=PN; 4.5=NA → Low. Direct time, distance, power, jump, or force outcomes were recorded with the same protocol/device; assessor judgment was unlikely to alter the recorded value.	5.1=NI; 5.2=PN; 5.3=NI; 5.4=NA → Some concerns. No analysis plan finalized before unblinding was verifiable; no clear selective omission was found, but analysis-choice selection could not be excluded.	High. Least-favourable domain rule.
**Ferrauti et al. (1997) (Crossover)**	1.1=NI; 1.2=NI; 1.3=NI → Some concerns. A double-blind counterbalanced pattern was reported, but random sequence generation and concealment were not.	S1=PY; S2=NA; S3=PY → Low. Counterbalanced sessions were separated by at least 3 days (pp. 4–5).	2.1=PN; 2.2=PN; 2.3=PN; 2.4=NA; 2.5=NA; 2.6=PY; 2.7=NA → Low. Double-blind administration and standardized sessions; no trial-context deviations were reported.	3.1=PY; 3.2–3.4=NA → Low. Eligible performance outcomes were available for all or nearly all participants.	4.1=PN; 4.2=PN; 4.3=PN; 4.4=PN; 4.5=NA → Low. Direct time, distance, power, jump, or force outcomes were recorded with the same protocol/device; assessor judgment was unlikely to alter the recorded value.	5.1=NI; 5.2=PN; 5.3=NI; 5.4=N → Some concerns. No analysis plan finalized before unblinding was verifiable; no clear selective omission was found, but analysis-choice selection could not be excluded.	Some concerns. Least-favourable domain rule.
**Millard-Stafford et al. (2005) (Crossover)**	1.1=NI; 1.2=NI; 1.3=NI → Some concerns. Counterbalancing was reported, but random sequence generation and allocation concealment were not.	S1=PY; S2=NA; S3=PY → Low. Counterbalanced tests were separated by at least 14 days; no trial-order effect was detected (p. 3).	2.1=PN; 2.2=PN; 2.3=PN; 2.4=NA; 2.5=NA; 2.6=PY; 2.7=NA → Low. Double-blind administration; no trial-context deviations were reported.	3.1=PY; 3.2–3.4=NA → Low. Eligible run-performance results were available for all or nearly all trial periods.	4.1=PN; 4.2=PN; 4.3=PN; 4.4=PN; 4.5=NA → Low. Direct time, distance, power, jump, or force outcomes were recorded with the same protocol/device; assessor judgment was unlikely to alter the recorded value.	5.1=NI; 5.2=PN; 5.3=NI; 5.4=N → Some concerns. No analysis plan finalized before unblinding was verifiable; no clear selective omission was found, but analysis-choice selection could not be excluded.	Some concerns. Least-favourable domain rule.

Study / design	D1 Randomization	DS Period / carryover	D2 Deviations / analysis	D3 Missing outcome	D4 Outcome measurement	D5 Selection of result	Overall
**Johannsen et al. (2013) (Crossover)**	1.1=PY; 1.2=NI; 1.3=NI → Some concerns. Randomized counterbalanced crossover was reported, but allocation concealment was not described.	S1=PY; S2=NA; S3=PY → Low. Counterbalanced sessions were separated by at least 2 weeks in the registry (NCT01893853).	2.1=PN; 2.2=PN; 2.3=PN; 2.4=NA; 2.5=NA; 2.6=PY; 2.7=NA → Low. Double-blind administration; no trial-context deviations were reported.	3.1=PY; 3.2–3.4=NA → Low. The eligible 30-min performance result was reported for the analyzed crossover sample.	4.1=PN; 4.2=PN; 4.3=PN; 4.4=PN; 4.5=NA → Low. Direct time, distance, power, jump, or force outcomes were recorded with the same protocol/device; assessor judgment was unlikely to alter the recorded value.	5.1=NI; 5.2=PN; 5.3=NI; 5.4=N → Some concerns. No analysis plan finalized before unblinding was verifiable; no clear selective omission was found, but analysis-choice selection could not be excluded.	Some concerns. Least-favourable domain rule.
**Davis et al. (1988) (Crossover)**	1.1=NI; 1.2=NI; 1.3=NI → Some concerns. Double-blind counterbalancing was reported, but random sequence generation and concealment were not.	S1=PY; S2=NA; S3=PY → Low. Counterbalanced trials were 4–7 days apart (report pp. 2–3).	2.1=PN; 2.2=PN; 2.3=PN; 2.4=NA; 2.5=NA; 2.6=PY; 2.7=NA → Low. Double-blind administration and repeated-measures analysis; no trial-context deviations were reported.	3.1=PY; 3.2–3.4=NA → Low. Eligible completion-time data were available for all or nearly all trial periods.	4.1=PN; 4.2=PN; 4.3=PN; 4.4=PN; 4.5=NA → Low. Direct time, distance, power, jump, or force outcomes were recorded with the same protocol/device; assessor judgment was unlikely to alter the recorded value.	5.1=NI; 5.2=PN; 5.3=NI; 5.4=N → Some concerns. No analysis plan finalized before unblinding was verifiable; no clear selective omission was found, but analysis-choice selection could not be excluded.	Some concerns. Least-favourable domain rule.
**Gant et al. (2007) (Crossover)**	1.1=PY; 1.2=NI; 1.3=NI → Some concerns. Random treatment order was reported, but the sequence method and concealment were not.	S1=NI; S2=NI; S3=PY → Some concerns. Visits were 7 days apart, but sequence balance was not reported (p. 3).	2.1=PN; 2.2=PN; 2.3=PN; 2.4=NA; 2.5=NA; 2.6=PY; 2.7=NA → Low. Double-blind drinks and standardized protocol; no trial-context deviations were reported.	3.1=PY; 3.2–3.4=NA → Low. Eligible sprint outcomes were available for all or nearly all periods.	4.1=PN; 4.2=PN; 4.3=PN; 4.4=PN; 4.5=NA → Low. Direct time, distance, power, jump, or force outcomes were recorded with the same protocol/device; assessor judgment was unlikely to alter the recorded value.	5.1=NI; 5.2=PN; 5.3=NI; 5.4=N → Some concerns. No analysis plan finalized before unblinding was verifiable; no clear selective omission was found, but analysis-choice selection could not be excluded.	Some concerns. Least-favourable domain rule.
**Bergen et al. (1998) (Crossover)**	1.1=PY; 1.2=NI; 1.3=NI → Some concerns. Random order was assigned by an independent research assistant who held the drink code until both trials were complete, but the report did not establish that the allocation sequence was unavailable to enrolling or assigning personnel before enrolment and assignment (thesis p. 53).	S1=NI; S2=NI; S3=PY → Some concerns. Long separation made carryover unlikely, but the allocation to treatment sequences was not reported as balanced.	2.1=PN; 2.2=PN; 2.3=PN; 2.4=NA; 2.5=NA; 2.6=PY; 2.7=NA → Low. The independent code holder supported double blinding; no trial-context deviations were reported.	3.1=PY; 3.2–3.4=NA → Low. Eligible termination-time outcomes were available for all or nearly all participants.	4.1=PN; 4.2=PN; 4.3=PN; 4.4=PN; 4.5=NA → Low. Direct time, distance, power, jump, or force outcomes were recorded with the same protocol/device; assessor judgment was unlikely to alter the recorded value.	5.1=NI; 5.2=PN; 5.3=NI; 5.4=N → Some concerns. No analysis plan finalized before unblinding was verifiable; no clear selective omission was found, but analysis-choice selection could not be excluded.	Some concerns. Least-favourable domain rule.

Study / design	D1 Randomization	DS Period / carryover	D2 Deviations / analysis	D3 Missing outcome	D4 Outcome measurement	D5 Selection of result	Overall
**Millard-Stafford et al. (1997) (Crossover)**	1.1=NI; 1.2=NI; 1.3=NI → Some concerns. Counterbalanced treatment order was reported, but random generation and allocation concealment were not.	S1=PY; S2=NA; S3=PY → Low. Runs were separated by at least 1 week, counterbalanced, and showed no test-order difference (PDF pp. 3–9).	2.1=PY; 2.2=NI; 2.3=PN; 2.4=NA; 2.5=NA; 2.6=PY; 2.7=NA → Low. Water was distinguishable from caloric drinks, but no trial-context deviations were reported; paired analysis followed assigned condition.	3.1=PY; 3.2–3.4=NA → Low. All 12 participants contributed eligible run-performance results.	4.1=PN; 4.2=PN; 4.3=NI; 4.4=PN; 4.5=NA → Low. Direct time, distance, power, jump, or force outcomes were recorded with the same protocol/device; assessor judgment was unlikely to alter the recorded value.	5.1=NI; 5.2=PN; 5.3=NI; 5.4=N → Some concerns. No analysis plan finalized before unblinding was verifiable; no clear selective omission was found, but analysis-choice selection could not be excluded.	Some concerns. Least-favourable domain rule.
**Gusbakti et al. (2006) (Crossover)**	1.1=PY; 1.2=NI; 1.3=NI → Some concerns. Random order was reported, but sequence generation and concealment were not described.	S1=NI; S2=NI; S3=PY → Some concerns. Trials were 2 weeks apart, but sequence balance was not reported (p. 2).	2.1=PN; 2.2=PN; 2.3=PN; 2.4=NA; 2.5=NA; 2.6=PY; 2.7=NA → Low. Double-blind administration; no trial-context deviations were reported.	3.1=PY; 3.2–3.4=NA → Low. Eligible time-to-exhaustion data were available for all or nearly all participants.	4.1=PN; 4.2=PN; 4.3=PN; 4.4=PN; 4.5=NA → Low. Direct time, distance, power, jump, or force outcomes were recorded with the same protocol/device; assessor judgment was unlikely to alter the recorded value.	5.1=NI; 5.2=PN; 5.3=NI; 5.4=N → Some concerns. No analysis plan finalized before unblinding was verifiable; no clear selective omission was found, but analysis-choice selection could not be excluded.	Some concerns. Least-favourable domain rule.
**Atan et al. (2024) (Crossover)**	1.1=PY; 1.2=NI; 1.3=NI → Some concerns. Randomized order was reported, but the sequence method and allocation concealment were not.	S1=NI; S2=NI; S3=PY → Some concerns. Trials were 7 days apart; sequence balance was not reported (pp. 1, 4).	2.1=PN; 2.2=PN; 2.3=PN; 2.4=NA; 2.5=NA; 2.6=PY; 2.7=NA → Low. Double-blind drinks; no trial-context deviations were reported.	3.1=PY; 3.2–3.4=NA → Low. Performance outcomes were complete; reduced blood sampling was not relevant to the meta-analyzed performance result.	4.1=PN; 4.2=PN; 4.3=PN; 4.4=PN; 4.5=NA → Low. Direct time, distance, power, jump, or force outcomes were recorded with the same protocol/device; assessor judgment was unlikely to alter the recorded value.	5.1=NI; 5.2=PN; 5.3=NI; 5.4=N → Some concerns. No analysis plan finalized before unblinding was verifiable; no clear selective omission was found, but analysis-choice selection could not be excluded.	Some concerns. Least-favourable domain rule.
**Morris et al. (2003) (Crossover)**	1.1=PY; 1.2=NI; 1.3=NI → Some concerns. Random counterbalanced order was reported, but allocation concealment was not.	S1=Y; S2=NA; S3=PY → Low. The design was explicitly balanced and trials were 7–10 days apart; the observed period trend cancels under balanced sequences (pp. 2, 9).	2.1=PN; 2.2=NI; 2.3=PN; 2.4=NA; 2.5=NA; 2.6=PY; 2.7=NA → Low. Taste-matched drinks and standardized testing; no trial-context deviations were reported.	3.1=PY; 3.2–3.4=NA → Low. Eligible distance and sprint outcomes were available for all or nearly all periods.	4.1=PN; 4.2=PN; 4.3=NI; 4.4=PN; 4.5=NA → Low. Direct time, distance, power, jump, or force outcomes were recorded with the same protocol/device; assessor judgment was unlikely to alter the recorded value.	5.1=NI; 5.2=PN; 5.3=NI; 5.4=N → Some concerns. No analysis plan finalized before unblinding was verifiable; no clear selective omission was found, but analysis-choice selection could not be excluded.	Some concerns. Least-favourable domain rule.

Study / design	D1 Randomization	DS Period / carryover	D2 Deviations / analysis	D3 Missing outcome	D4 Outcome measurement	D5 Selection of result	Overall
**Murray et al. (1989) (Crossover)**	1.1=NI; 1.2=NI; 1.3=NI → Some concerns. Double-blind counterbalancing was reported, but random sequence generation and concealment were not.	S1=PY; S2=NA; S3=PY → Low. Counterbalanced tests were separated by 7–10 days (report pp. 2–3).	2.1=PN; 2.2=PN; 2.3=PN; 2.4=NA; 2.5=NA; 2.6=PY; 2.7=NA → Low. Double-blind drinks and standardized testing; no trial-context deviations were reported.	3.1=PY; 3.2–3.4=NA → Low. Eligible time-trial results were available for all or nearly all participants.	4.1=PN; 4.2=PN; 4.3=PN; 4.4=PN; 4.5=NA → Low. Direct time, distance, power, jump, or force outcomes were recorded with the same protocol/device; assessor judgment was unlikely to alter the recorded value.	5.1=NI; 5.2=PN; 5.3=NI; 5.4=N → Some concerns. No analysis plan finalized before unblinding was verifiable; no clear selective omission was found, but analysis-choice selection could not be excluded.	Some concerns. Least-favourable domain rule.
**Millard-Stafford et al. (1992) (Crossover)**	1.1=NI; 1.2=NI; 1.3=NI → Some concerns. Counterbalanced double-blind design was reported, but random sequence generation and concealment were not.	S1=PY; S2=NA; S3=PY → Low. Counterbalanced trials were separated by a mean of 15 days (PDF pp. 1–2).	2.1=PN; 2.2=PN; 2.3=PN; 2.4=NA; 2.5=NA; 2.6=PY; 2.7=NA → Low. Double-blind administration; no trial-context deviations were reported.	3.1=N; 3.2=N; 3.3=Y; 3.4=Y → High. Two of eight participants did not complete 40 km under placebo; their times were extrapolated from the last completed 5-km segment, so missingness depended on poor performance and treatment (PDF p. 4).	4.1=PN; 4.2=PN; 4.3=PN; 4.4=PN; 4.5=NA → Low. Direct time, distance, power, jump, or force outcomes were recorded with the same protocol/device; assessor judgment was unlikely to alter the recorded value.	5.1=NI; 5.2=PN; 5.3=NI; 5.4=N → Some concerns. No analysis plan finalized before unblinding was verifiable; no clear selective omission was found, but analysis-choice selection could not be excluded.	High. Least-favourable domain rule.
**Byrne et al. (2005) (Crossover)**	1.1=PY; 1.2=NI; 1.3=NI → Some concerns. A randomized crossover was reported, but sequence generation and allocation concealment were not described.	S1=NI; S2=NI; S3=NI → Some concerns. Neither sequence balance nor the between-period interval was reported (p. 2).	2.1=PY; 2.2=NI; 2.3=PN; 2.4=NA; 2.5=NA; 2.6=PY; 2.7=NA → Low. Water and sports drink were distinguishable, but no trial-context deviations were reported and paired analysis followed assignment.	3.1=PY; 3.2–3.4=NA → Low. Eligible marching-duration data were available for all or nearly all participants.	4.1=PN; 4.2=PN; 4.3=NI; 4.4=PN; 4.5=NA → Low. Direct time, distance, power, jump, or force outcomes were recorded with the same protocol/device; assessor judgment was unlikely to alter the recorded value.	5.1=NI; 5.2=PN; 5.3=NI; 5.4=N → Some concerns. No analysis plan finalized before unblinding was verifiable; no clear selective omission was found, but analysis-choice selection could not be excluded.	Some concerns. Least-favourable domain rule.
**Millard-Stafford et al. (1990) (Crossover)**	1.1=NI; 1.2=NI; 1.3=NI → Some concerns. Counterbalanced double-blind order was reported, but random sequence generation and concealment were not.	S1=PY; S2=NA; S3=PY → Low. Counterbalanced triathlon tests were separated by at least 7 days (PDF p. 2).	2.1=PN; 2.2=PN; 2.3=PN; 2.4=NA; 2.5=NA; 2.6=PY; 2.7=NA → Low. Double-blind administration; no trial-context deviations were reported.	3.1=PY; 3.2–3.4=NA → Low. All 10 participants contributed both-condition performance results.	4.1=PN; 4.2=PN; 4.3=PN; 4.4=PN; 4.5=NA → Low. Direct time, distance, power, jump, or force outcomes were recorded with the same protocol/device; assessor judgment was unlikely to alter the recorded value.	5.1=NI; 5.2=PN; 5.3=NI; 5.4=N → Some concerns. No analysis plan finalized before unblinding was verifiable; no clear selective omission was found, but analysis-choice selection could not be excluded.	Some concerns. Least-favourable domain rule.

Study / design	D1 Randomization	DS Period / carryover	D2 Deviations / analysis	D3 Missing outcome	D4 Outcome measurement	D5 Selection of result	Overall
**Bilzon et al. (2000) (Crossover)**	1.1=NI; 1.2=NI; 1.3=NI → Some concerns. Double-blind crossover was reported, but random order, sequence balance, and concealment were not.	S1=NI; S2=NI; S3=PY → Some concerns. Trials were at least 7 days apart, but sequence balance and period-effect handling were not reported (PDF pp. 1–2).	2.1=PN; 2.2=PN; 2.3=PN; 2.4=NA; 2.5=NA; 2.6=PY; 2.7=NA → Low. Double-blind administration; no trial-context deviations were reported.	3.1=PY; 3.2–3.4=NA → Low. All 13 participants completed both conditions and the eligible time-to-exhaustion outcome.	4.1=PN; 4.2=PN; 4.3=PN; 4.4=PN; 4.5=NA → Low. Direct time, distance, power, jump, or force outcomes were recorded with the same protocol/device; assessor judgment was unlikely to alter the recorded value.	5.1=NI; 5.2=PN; 5.3=NI; 5.4=N → Some concerns. No analysis plan finalized before unblinding was verifiable; no clear selective omission was found, but analysis-choice selection could not be excluded.	Some concerns. Least-favourable domain rule.

Response codes: Y, yes; PY, probably yes; PN, probably no; N, no; NI, no information; NA, not applicable. Judgments refer to eligible direct-performance results; where a study contributed more than one eligible result, the displayed study-level judgment is the least favourable. NI was not converted to a favourable response merely because a report used a generic blinding label.

Signalling-question key: D1—1.1 random sequence, 1.2 allocation concealed, 1.3 baseline imbalance; for the cluster-assigned trial, the cluster-randomized addendum also assessed whether participant identification or recruitment timing could introduce bias. DS—S1 sequence balance, S2 period effects modelled if required, S3 sufficient time for carryover to disappear; D2—2.1 participant awareness, 2.2 personnel awareness, 2.3 trial-context deviations, 2.4 likely effect on outcome, 2.5 balanced deviations, 2.6 appropriate analysis, 2.7 likely substantial impact; D3—3.1 data complete, 3.2 evidence result not biased, 3.3 missingness could depend on true value, 3.4 likely dependence; D4—4.1 appropriate method, 4.2 comparable method, 4.3 assessor awareness, 4.4 assessment could be influenced, 4.5 likely influence; D5—5.1 prespecified plan, 5.2 multiple measurements, 5.3 multiple analyses, 5.4 first-period-only result after identified carryover.

Supplementary Material S10 (Time-Trial and Time-to-Exhaustion Outcome-Type Analyses)

Analysis	Outcome category	Studies	K	Hedges' g [95% CI]	p value	95% PI	I^2 total	Between-category p
Strict post hoc comparison	Time-trial/fixed-work completion	7	12	0.41 [0.12, 0.69]	0.014	[-0.11, 0.92]	26.21%	0.303
Strict post hoc comparison	Explicit time to exhaustion	5	6	0.66 [0.06, 1.26]	0.038	[-0.57, 1.89]	59.89%	
Broad sensitivity analysis	Time-trial/fixed-work performance	8	13	0.40 [0.16, 0.64]	0.005	[0.01, 0.79]	16.39%	0.668
Broad sensitivity analysis	Time-to-exhaustion/exercise capacity	7	8	0.49 [0.01, 0.97]	0.048	[-0.66, 1.64]	65.29%	

Notes: Post hoc analyses within the aerobic-endurance domain. The strict comparison excludes outcomes that do not meet either protocol definition; the broad sensitivity analysis reassigns those outcomes as described below. K, number of effect sizes; CI, confidence interval; PI, prediction interval. Strict time-trial/fixed-work completion required a fixed amount of work or distance completed as quickly as possible; explicit TTE required an outcome reported as time to exhaustion. Three aerobic outcomes were excluded from the strict comparison but retained in the full aerobic-domain model: 30-minute total work (Johannsen et al.), distance during the LIST protocol with multiple stopping criteria (Morris et al.), and cumulative loaded-marching duration (Byrne et al.). The broad sensitivity analysis assigned Johannsen et al. to performance-type tests and Morris et al. and Byrne et al. to capacity-to-termination tests. All estimates were fitted with three-level REML models using the corrected effect sizes, the original sampling variance–covariance structure, $r_{pair} = 0.50$, and $\rho = 0.50$. Between-category p values are from mixed-effects moderator tests. These analyses were added post hoc and should be interpreted as exploratory.

Supplementary Material S11 (Ambient-Temperature Threshold Sensitivity Analyses)

Temperature criterion	Studies	K	N	Hedges' g [95% CI]	p value	95% PI	I² total	Status
≥22 °C (primary operational boundary)	21	32	316	0.371 [0.214, 0.528]	<0.001	[−0.214, 0.956]	46.7%	Operational eligibility boundary; secondary omnibus synthesis
>25 °C (22–25 °C excluded)	18	27	273	0.410 [0.228, 0.592]	<0.001	[−0.248, 1.068]	51.2%	Post hoc
≥28 °C	13	19	188	0.359 [0.108, 0.609]	0.009	[−0.473, 1.190]	58.3%	Post hoc

Notes: Post hoc threshold sensitivity analyses addressed the operational ⩾22 °C lower eligibility boundary. The same corrected three-level REML model and variance–covariance structure (paired-condition r_pair = 0.50; within-study dependence ρ = 0.50) were refitted after excluding studies conducted at 22.0–25.0 °C (>25 °C subset) and after applying a stricter ⩾28 °C threshold. The ⩾28 °C restriction was selected as a stricter published operational criterion for exercise in hot environments, not as a universal physiological threshold. Hsu et al. (2026) was excluded from the review because neither quantitative ambient temperature nor WBGT was reported sufficiently to verify the operational ⩾22 °C eligibility boundary. This table reports the secondary omnibus direct-performance synthesis under each temperature restriction. K, number of effect sizes; N, unique participants; CI, confidence interval; PI, prediction interval. The positive mean direction was retained under both higher-temperature restrictions, but both prediction intervals included zero and precision decreased as the evidence base became smaller. These analyses test dependence on an operational eligibility boundary and do not establish a heat-specific protective effect.

Supplementary Material S12 (Conservative PEDro Checklist Audit; Descriptive Only)

Study	I1†	I2	I3	I4	I5	I6	I7	I8	I9	I10	I11	Total/10
O'Neal et al. (2013)	Y	Y	NR	NR	N	N	NR	Y	Y	Y	Y	5
Kalman et al. (2012)	Y	Y	NR	NR	N	N	NR	Y	N	Y	Y	4
Lee et al. (2011)	Y	Y	NR	NR	N	N	NR	Y	Y	Y	Y	5
Del Coso et al. (2008)	Y	Y	NR	NR	N	NR	NR	Y	Y	Y	Y	5
Liang et al. (2024)	Y	Y	NR	NR	N	NR	NR	Y	N	Y	Y	4
Criswell et al. (1991)	Y	Y	NR	NR	Y	NR	NR	Y	Y	Y	Y	6
Ferrauti et al. (1997)	Y	NR	NR	NR	Y	NR	NR	Y	Y	Y	Y	5
Millard-Stafford et al. (2005)	Y	NR	NR	NR	Y	NR	NR	Y	Y	Y	Y	5
Johannsen et al. (2013)	Y	Y	NR	NR	N	NR	NR	Y	Y	Y	Y	5
Davis et al. (1988)	Y	NR	NR	NR	Y	NR	NR	Y	Y	Y	Y	5
Gant et al. (2007)	Y	Y	NR	NR	Y	NR	NR	Y	Y	Y	Y	6
Bergen et al. (1998)	Y	Y	NR	NR	Y	NR	NR	Y	Y	Y	Y	6
Millard-Stafford et al. (1997)	Y	NR	NR	NR	N	N	NR	Y	Y	Y	Y	4
Gusbakti et al. (2006)	Y	Y	NR	NR	NR	NR	NR	Y	Y	Y	Y	5
Atan et al. (2024)	Y	Y	NR	NR	Y	NR	NR	Y	Y	Y	Y	6
Morris et al. (2003)	Y	Y	NR	NR	Y	NR	NR	Y	Y	Y	Y	6
Murray et al. (1989)	Y	NR	NR	NR	Y	NR	NR	Y	Y	Y	Y	5
Millard-Stafford et al. (1992)	Y	NR	NR	NR	Y	NR	NR	N	Y	Y	Y	4
Byrne et al. (2005)	Y	Y	NR	NR	N	N	NR	Y	Y	Y	Y	5
Millard-Stafford et al. (1990)	Y	NR	NR	NR	Y	NR	NR	Y	Y	Y	Y	5
Bilzon et al. (2000)	Y	NR	NR	NR	NR	NR	NR	NR	NR	Y	Y	2

Item key: I1, eligibility criteria specified (not scored); I2, random allocation/order; I3, allocation concealment; I4, baseline comparability; I5, participant blinding; I6, provider/therapist blinding; I7, assessor blinding; I8, >85% key-outcome follow-up; I9, received allocated condition or intention-to-treat analysis; I10, between-group/condition comparison; I11, point estimate and variability.

Notes: PEDro formally awards a point (yes) or no point. Y means clearly met. N means the criterion was not met. NR is used here only as an audit-transparency label for insufficient reporting; it is not an official third PEDro response category and receives no point. † Item 1 is recorded but excluded from the 10-point total. Crossover design did not automatically satisfy baseline comparability. Generic 'double-blind' wording was not accepted as evidence of provider/therapist or assessor blinding without role-specific reporting. Revised totals: range 2–6, mean 4.9, median 5.

Supplementary Material S13 (Data-Source Provenance Audit)

AUDIT ITEM	DOCUMENTED COUNT	STUDY/EFFECT	RESOLUTION
AUTHOR CONTACTS	4 studies	Missing or unclear numerical information	Authors were contacted for clarification or data.
SUCCESSFUL AUTHOR REPLIES	2 studies	Author-provided information	Replies were incorporated where relevant and cross-checked.
WEBPLOTDIGITIZER	4 outcome datasets	Graphically reported numerical outcomes	Values were independently cross-checked by two reviewers.
OTHER FINAL EFFECTS	Remaining final effect sizes	All other included effects	Extracted from numerical reports or reproducible transformations.

Note: Counts of author contacts, successful replies, and digitized datasets were confirmed by the corresponding author for this revision. Digitized values were checked against the original figures by two reviewers.
